# Hydrophobicity Tailoring of Ferric Covalent Organic Framework/MXene Nanosheets for High‐Efficiency Nitrogen Electroreduction to Ammonia

**DOI:** 10.1002/advs.202206933

**Published:** 2023-03-30

**Authors:** Hongming He, Hao‐Ming Wen, Hong‐Kai Li, Ping Li, Jiajun Wang, Yijie Yang, Cheng‐Peng Li, Zhihong Zhang, Miao Du

**Affiliations:** ^1^ College of Chemistry Tianjin Key Laboratory of Structure and Performance for Functional Molecules Tianjin Normal University Tianjin 300387 China; ^2^ College of Material and Chemical Engineering Institute of New Energy Science and Technology School of Future Hydrogen Energy Technology Zhengzhou University of Light Industry Zhengzhou 450001 China

**Keywords:** 2D materials, electrochemical nitrogen fixation, ferric covalent organic frameworks, hydrophobicity, MXenes

## Abstract

Electrocatalytic nitrogen reduction reaction (NRR) represents a promising sustainable approach for NH_3_ synthesis. However, the poor NRR performance of electrocatalysts is a great challenge at this stage, mainly owing to their low activity and the competitive hydrogen evolution reaction (HER). Herein, 2D ferric covalent organic framework/MXene (COF‐Fe/MXene) nanosheets with controllable hydrophobic behaviors are successfully prepared via a multiple‐in‐one synthetic strategy. The boosting hydrophobicity of COF‐Fe/MXene can effectively repel water molecules to inhibit the HER for enhanced NRR performances. By virtue of the ultrathin nanostructure, well‐defined single Fe sites, nitrogen enrichment effect, and high hydrophobicity, the 1H,1H,2H,2H‐perfluorodecanethiol modified COF‐Fe/MXene hybrid shows a NH_3_ yield of 41.8 µg h^−1^ mg_cat._^−1^ and a Faradaic efficiency of 43.1% at −0.5 V versus RHE in a 0.1 m Na_2_SO_4_ water solution, which are vastly superior to the known Fe‐based catalysts and even to the noble metal catalysts. This work provides a universal strategy to design and synthesis of non‐precious metal electrocatalysts for high‐efficiency N_2_ reduction to NH_3_.

## Graphical abstract

COF‐Fe/MXene nanosheets are designed and prepared for electrocatalytic N_2_ reduction to NH_3_. Especially, the controllable hydrophobic properties of electrocatalysts can remarkably improve the nitrogen reduction reaction performances via inhibiting the competitive hydrogen evolution reaction at ambient conditions. This work opens up a universal approach to construct non‐precious metal electrocatalysts for ambient N_2_ fixation.

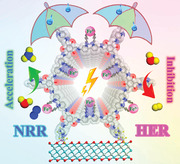

## 1 Introduction

Ammonia (NH_3_) is an essential feedstock for nitrogen‐based fertilizers and pharmaceuticals.^[^ ^1^ ^]^ Nowadays, the unsustainable Haber–Bosch technique is extensively utilized to synthesize over 90% NH_3_ worldwide. Nevertheless, the extreme inertia of N_2_ causes harsh reaction conditions (400–450 °C and 15–25 MPa), leading to tremendous energy consumption and CO_2_ emission.^[^ ^2^ ^]^ It is urgent to develop eco‐friendly and sustainable approaches for NH_3_ synthesis under ambient conditions. Electrocatalytic nitrogen reduction reaction (NRR), as an energy conservation and environmental method for ammonia synthesis, has recently attracted considerable attentions.^[^ ^3^ ^]^ Multifarious electrocatalysts have been designed and used for N_2_ reduction to NH_3_ so far, such as noble metals,^[^ ^4^ ^]^ non‐noble metals,^[^ ^5^ ^]^ alloys,^[^ ^6^ ^]^ and non‐metallic materials.^[^ ^7^ ^]^ Disappointingly, their poor catalytic performances are insufficient to meet the actual requirement due to the high N≡N triple bond energy (940.95 kJ mol^−1^) and the severe hydrogen evolution reaction (HER).^[^ ^8^ ^]^ Therefore, the development of high‐performance NRR electrocatalysts, especially those of non‐noble metals, are in urgent need.

Up to now, numerous covalent organic frameworks (COFs) have been rationally constructed by covalent linkage of organic building primitives.^[^ ^9^ ^]^ By virtue of their large surface areas, well‐defined porosity, precise skeleton structures, adjustable porous environments, and high stability, COFs are greatly promising substrate materials to load metal catalytic sites.^[^ ^10^ ^]^ Meanwhile, the hydrophobicity of COFs can be controlled by introducing hydrophobic molecules or functional groups.^[^ ^11^ ^]^ Thus, the hydrophobic and metallized COFs may be beneficial to N_2_ electroreduction to NH_3_ via synergistic effect between the highly active metal sites and the effective suppression of HER. However, the conductivity of COFs is normally poor to greatly weaken their activities for electrocatalytic reactions, though some conductive COFs have been explored to this aim.^[^ ^12^ ^]^ Remarkably, MXenes, as 2D transition metal nitrides/carbides/carbonitrides, are promising to construct extended architectures due to their excellent electronic conductivity, charge mobility anisotropy, and tunable surface terminated groups,^[^ ^13^ ^]^ which have been widely used to support various functional materials.^[^ ^14^ ^]^ When MXene is modified by highly active organic groups on surface, COFs can be uniformly and tightly integrated with MXenes to form heterojunction nanostructures via covalent coupling.^[^ ^15^ ^]^ Moreover, 2D metallized COF/MXene nanosheets may further promote the electrocatalytic NRR, for their large aspect‐ratios, accessible active sites, effective mass transfer, and high dispersibility.^[^ ^16^ ^]^ Therefore, the rational assembly of COF and MXene nanosheets in a 2D–2D format with tailored hydrophobic properties is anticipated to provide a promising platform for electrocatalytic N_2_ fixation.

**Scheme 1:**
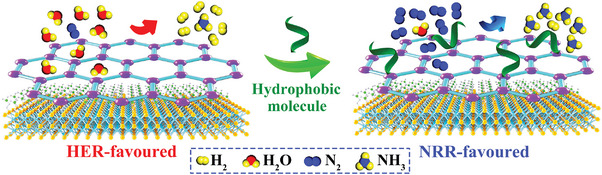
Schematic illustration of hydrophobic COF/MXene nanosheets for electrocatalytic N_2_‐to‐NH_3_ transformation.

To confirm this proof‐of‐concept, we develop an executable synthetic strategy to prepare 2D metallized COF/MXene nanosheets with controllable hydrophobicity for electrocatalytic NRR (**Scheme** **1**). Ti_3_C_2_T*_x_* MXene nanosheets with —NH_2_ moieties can promote the in situ growth of COFs via interfacial synthesis. Considering the composition and mechanism of nitrogenase, the Fe element is a perfect non‐noble metal candidate for N_2_‐to‐NH_3_ fixation.^[^ ^17^ ^]^ In this work, the as‐synthesized COF/MXene nanosheet is post‐processed in ferric trichloride methanol solution to generate COF‐Fe/MXene, which is further decorated with different functional molecules to obtain hydrophobic COF‐Fe/MXene.^[^ ^18^ ^]^ These hydrophobic hybrids can catalyze N_2_ to NH_3_ in Na_2_SO_4_ solution efficiently due to their ultrathin nanosheets, high stability, well‐defined single Fe sites, N_2_ concentration effect, and hydrophobicity. Notably, the increased hydrophobicity of the series of metallized (Fe, Co, Ni, and Cu) COF/MXene electrocatalysts can greatly suppress the competitive HER and boost the N_2_ reduction to NH_3_.

## 2 Results and Discussion

**Scheme 2:**
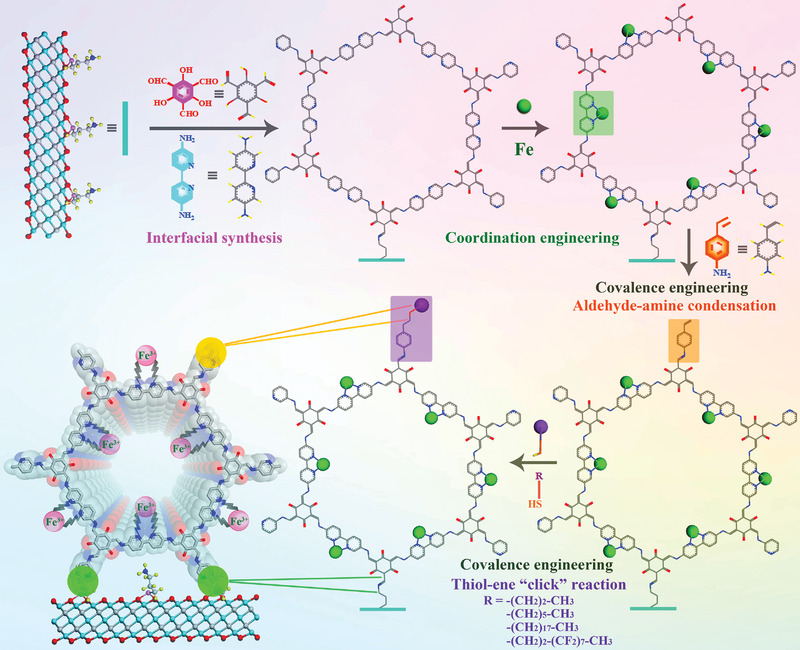
Schematic diagram for the synthesis of hydrophobic COF‐Fe/MXene nanosheets.

The synthetic illustration of COF‐Fe/MXene nanosheets with hydrophobic group is depicted in **Scheme** **2**. The amino‐functionalized Ti_3_C_2_T*_x_* MXene was ultrasonically dispersed in water and 2,2′‐bipyridine‐5,5′‐diamine (Bpy) was dissolved in a mixture of acetonitrile and water, which were slowly added on a dichloromethane solution of 1,3,5‐triformylphloroglucinol (Tp). By the interfacial crystallization synthesis method,^[^ ^19^ ^]^ COF/MXene nanosheets can be generated at the junction of solvents via Schiff base condensation. Notably, the highly active alkylamino groups of MXene are more favorable to generate imine bonds with the Tp monomers, leading to a rapid nucleation and growth of COF on MXene.^[^ ^20^ ^]^ Further, COF‐Fe/MXene hybrid was synthesized by immersing COF/MXene in a ferric trichloride methanol solution. Moreover, the hydrophobic alkyl molecules can be readily integrated on COF‐Fe/MXene via a thiol‐ene “click” reaction to obtain the hydrophobic COF‐Fe/MXene using 4‐aminostyrene as the binding agent.

**Figure 1:**
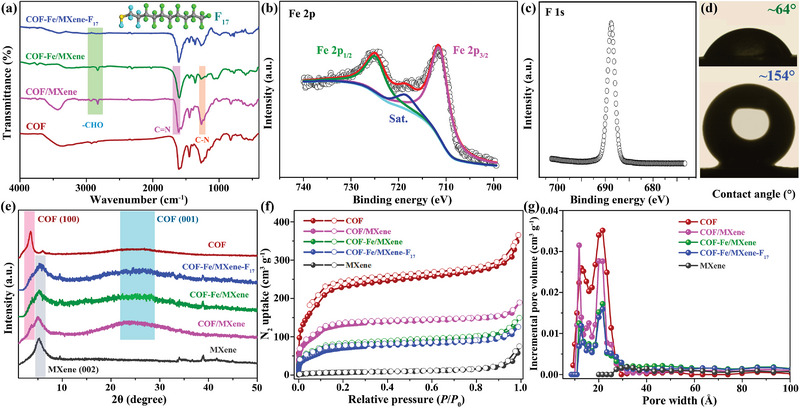
a) FT‐IR spectra. b) Fe 2p XPS of COF‐Fe/MXene. c) F 1s XPS of COF‐Fe/MXene‐F_17_. d) Water contact angle images of COF‐Fe/MXene (up) and COF‐Fe/MXene‐F_17_ (down). e) PXRD patterns, f) N_2_ sorption isotherms, and g) pore size distributions of different materials.

Fourier transform infrared (FT‐IR) and X‐ray photoelectron spectroscopy (XPS) indicate that 3‐aminopropyl triethoxysilane is covalently bonded to MXene, as testified by the N—H, Si—O, and C—H characteristic peaks of FT‐IR (Figure S1, Supporting Information) as well as N 1s, C—Si, and Si—O—Si signals of XPS (Figures S2 and S3, Supporting Information).^[^ ^21^ ^]^ The FT‐IR spectrum of COF/MXene is similar to that of COF except for a weak C—H characteristic peak of —CHO because of the incompletely reacted aldehyde group of Tp (**Figure** **1**a and Figure S4, Supporting Information).^[^ ^22^ ^]^ When the pristine MXene without terminal amino modification is assembled with COF, the obtained COF/MXene hybrid is irregular and inhomogeneous (Figure S5, Supporting Information). Thereby, the amino group on MXene plays an important role as a reactive cross‐linking site to construct the uniform COF/MXene heterostructure. The synthetic process of COF/MXene is verified by powder X‐ray diffraction (PXRD) and thermogravimetric analysis. The mass fraction of COF in COF/MXene hybrid gradually enhances with the extending reaction time and achieves the maximum COF amount of ≈36 wt% after 3 days, which are consistent with the PXRD results showing the emergence of COF characteristic peaks (Figures S6 and S7, Supporting Information). The optical images exhibit that the color of upper solution fades away after generating the thin film (Figure S8, Supporting Information). Notably, the Fe‐functionalized COF/MXene (COF‐Fe/MXene) and COF/MXene show slightly different C—N FT‐IR peaks and N 1s XPS signals on account of the coordination interactions between Fe ions and bipyridine N atoms of COFs (Figure 1a and Figure S9, Supporting Information).^[^ ^23^ ^]^ According to the previous reports,^[^ ^24^ ^]^ the ferric cation may be coordinated with two N atoms from one bipyridine ligand and three counter Cl^−^ anions from FeCl_3_ as confirmed by the high‐resolution Cl 2p spectrum (Figure S10, Supporting Information). The XPS characteristic peak of Fe 2p is only observed in COF/MXene, indicating the successful introduction of Fe (Figure 1b and Figure S11, Supporting Information). The high‐resolution Fe 2p spectrum of COF‐Fe/MXene has two peaks centered at ≈711.6 (Fe 2p_3/2_) and ≈725.0 eV (Fe 2p_1/2_) with a shakeup satellite peak at ≈718.6 eV, which reveals that only Fe^3+^ is present.^[^ ^25^ ^]^

The maximum Fe content of COF‐Fe/MXene is ≈2.5 wt%, as revealed by inductive coupled plasma‐optical emission spectrometer (ICP‐OES). The hydrophobicity is integrated onto COF‐Fe/MXene by using 1H,1H,2H,2H‐perfluorodecanethiol, resulting in COF‐Fe/MXene‐F_17_ with 4‐aminostyrene as cross‐linked reagent. New peaks of F 1s and S 2p appear in the XPS spectrum of COF‐Fe/MXene‐F_17_, compared to that of COF‐Fe/MXene (Figure 1c and Figures S12–S16, Supporting Information), evidently confirming the successful incorporation of 1H,1H,2H,2H‐perfluorodecanethiol and 4‐aminostyrene on COF‐Fe/MXene.^[^ ^26^ ^]^ Meanwhile, COF‐Fe/MXene‐F_17_ has the similar high‐resolution Fe 2p spectrum and the Fe content of ≈2.4 wt% with those of COF‐Fe/MXene, illustrating the same oxidative state and mass fraction of Fe^3+^ in both samples (Figure 1b and Figure S17, Supporting Information). The C—H characteristic peak of —CHO is not found in the FT‐IR spectrum of COF‐Fe/MXene‐F_17_ (Figure 1a), because the unreacted aldehyde forms amide bond with the —NH_2_ group of 4‐aminostyrene. Notably, COF‐Fe/MXene‐F_17_ cannot be synthesized without 4‐aminostyrene, as testified by none F signal in the XPS spectrum and unchanged hydrophobicity of the product, which confirm the necessity of this binding agent (Figures S18–S20, Supporting Information). The water droplet is absorbed on COF‐Fe/MXene with a static water contact angle of ≈64° due to the preferable hydrophilicity, but the water droplet can stand on the surface of COF‐Fe/MXene‐F_17_ with a significantly larger contact angle of ≈154° (Figure 1d), because the hydrophobic COF‐Fe/MXene‐F_17_ can repel the water. Compared with COF‐Fe/MXene, the as‐synthesized COF‐Fe/MXene‐F_17_ transfers from water to cyclohexane in the biphasic system because of its strong hydrophobicity (Figure S21, Supporting Information). In the PXRD patterns (Figure 1e), a broad peak of (002) crystal plane at ≈5.3° confirms the ordered stacking form of 2D MXene nanosheets with a d‐spacing of ≈1.67 nm based on the Bragg's law. The PXRD patterns further exhibit the (100) and (001) diffraction at ≈3.6° and ≈24.5° of COFs and the (002) lattice at ≈5.3° of MXene within COF/MXene, COF‐Fe/MXene, and COF‐Fe/MXene‐F_17_, revealing the successful synthesis of COF/MXene hybrids that maintain the original skeletons during the post‐modified process.^[^ ^27^ ^]^ N_2_ sorption isotherms indicate that the order of saturated adsorption amount is followed as COF nanosheet (≈366 cm^3^ g^−1^) > COF/MXene (≈189 cm^3^ g^−1^) > COF‐Fe/MXene (≈150 cm^3^ g^−1^) > COF‐Fe/MXene‐F_17_ (≈126 cm^3^ g^−1^) with a decreased Brunauer–Emmett–Teller surface area of 708, 426, 260, and 239 m^2^ g^−1^ caused by the introduction of nonporous MXene, ferric ion, and 1H,1H,2H,2H‐perfluorodecanethiol, respectively (Figure 1f). Notably, the pore size distributions indicate that these hybrids possess similar pore sizes with decreasing pore volumes (Figure 1g), illustrating that the thiol‐ene coupling reaction primarily occurs on COF‐Fe/MXene surface with accessible pores for N_2_ molecules.

**Figure 2:**
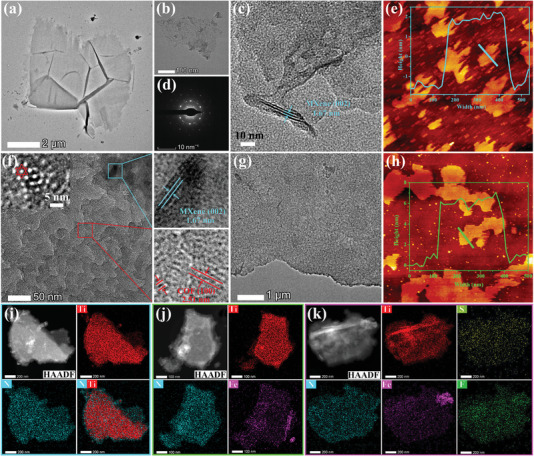
a–c) TEM, d) SAED, and e) AFM images of the amino‐functionalized MXene. TEM images of f) COF/MXene and g) COF‐Fe/MXene‐F_17_ with h) its AFM image. HAADF‐STEM and EDS elemental mappings of i) COF/MXene, j) COF‐Fe/MXene, and k) COF‐Fe/MXene‐F_17_.

Furthermore, the microarchitecture and layer thickness of hybrid materials were analyzed by transmission electron microscopy (TEM), selected area electron diffraction (SAED), scanning electron microscopy (SEM), and atomic force microscopy (AFM). As a result, the alkylamino group modified MXene is a 2D thin layer with the thickness of ≈3 nm and a lattice spacing of ≈1.67 nm for the (002) crystal plane of MXene (**Figure** **2**a–e). The SEM images of COF/MXene illustrate a 2D thin layer structure with the rough surface (Figure S22, Supporting Information). COF/MXene possesses the parallel‐stacked interplanar lattice of MXene and the crystal texture of COFs, with a (100) interplanar spacing of ≈2.51 nm as observed in the pristine COF (Figure 2f and Figure S23, Supporting Information). The morphologies of COF‐Fe/MXene and COF‐Fe/MXene‐F_17_ are similar to that of COF/MXene as illustrated by the TEM images (Figure 2g, Figures S24 and S25, Supporting Information). The AFM image of COF‐Fe/MXene‐F_17_ reveals that this hybrid is still a 2D nanosheet with the thickness of ≈6 nm (Figure 2h). The high‐angle annular dark field‐scanning transmission electron microscopy (HAADF‐STEM) with energy‐dispersive spectroscopy (EDS) elemental mappings demonstrate that the N and Ti elements are homogeneously distributed throughout the COF/MXene nanosheet (Figure 2i). The Ti element of MXene is not found at the edge of COF/MXene, but the N element covers a larger area than that of Ti, which indicates the growth of COF layers on MXene. The newly emerging Fe signal in COF‐Fe/MXene reveals the presence of Fe in the whole skeleton (Figure 2j). Also, HAADF‐STEM and EDS elemental mappings of COF‐Fe/MXene‐F_17_ exhibit the uniformly dispersed Ti, N, Fe, S, and F elements (Figure 2k), verifying the successful synthesis of COF‐Fe/MXene‐F_17_ heterostructures with the mass fraction of ≈3.1 wt% for F species.

**Figure 3:**
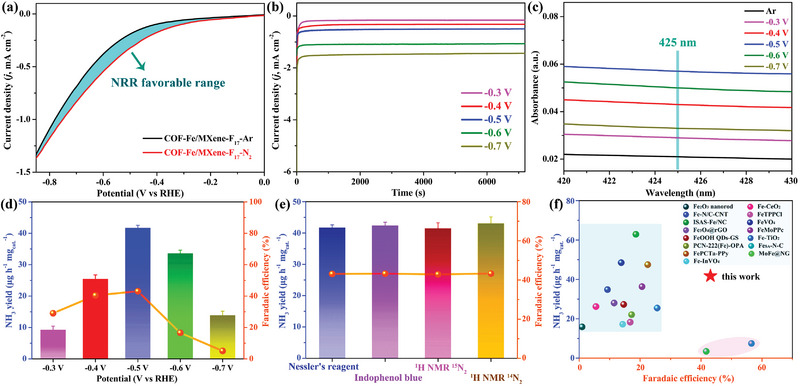
The catalytic performance of COF‐Fe/MXene‐F_17_: a) LSV curves under saturated Ar and N_2_ atmospheres. b) CA curves at different potentials. c) UV–vis absorption spectra of post‐electrolytes. d) NRR performances at different potentials. e) NRR performances determined by different methods. f) Electrocatalytic NRR performances for some representative Fe‐based catalysts.

Electrochemical measurements were performed in a 0.1 m Na_2_SO_4_ aqueous solution at room temperature. Different methods were used to quantitatively determine the NH_3_ yields, including ultraviolet visible (UV–vis) absorption spectroscopy^[^ ^28^ ^]^ and ^1^H NMR spectra (Figures S26–S30, Supporting Information).^[^ ^29^ ^]^ Compared with the current density under Ar atmosphere, the linear sweep voltammetry (LSV) curve of COF‐Fe/MXene‐F_17_ exhibits a higher current density in the N_2_‐saturated electrolyte during the NRR favorable range, confirming the extra NRR occurrence in the existence of N_2_ and COF‐Fe/MXene‐F_17_ (**Figure** **3**a). In addition, the reduction current of COF‐Fe/MXene‐F_17_ in Ar is mainly attributed to the HER, as confirmed by the cyclic voltammetry (CV) curves (Figure S31, Supporting Information). According to the main NRR range from −0.3 to −0.7 V versus the reversible hydrogen electrode (RHE), chronoamperometry (CA) curves of COF‐Fe/MXene‐F_17_ in the N_2_ atmosphere were measured at each given potential for 2 h separately. The CA curves suggest that the current density significantly increases from −0.3 to −0.7 V versus RHE without apparent time‐dependent change, which preliminarily confirms the possible electrocatalytic activity of COF‐Fe/MXene‐F_17_ at these potentials (Figure 3b). The electrolytes after NRR in the N_2_ condition were analyzed using the Nessler's reagent method, indicating the highest absorption spectrum at 425 nm with the maximum NH_3_ production yield of 41.8 µg h^−1^ mg_cat._^−1^ and the highest Faradaic efficiency of 43.1% at −0.5 V versus RHE, respectively (Figure 3c,d). The catalytic result has also been corroborated by using other quantitative methods, including indophenol blue and ^1^H NMR spectra (Figures S28–S30, Supporting Information). The similar results prove the accuracy and authenticity of quantitative analysis (Figure 3e) for NH_3_ synthesis. The electrocatalytic N_2_‐to‐NH_3_ fixation for some Fe‐based catalysts are summarized in Figure 3f and Table S1, Supporting Information.^[^ ^30^ ^]^ The prominent UV–vis absorption peak is only observed at −0.5 V versus RHE for COF‐Fe/MXene‐F_17_ in N_2_‐saturated atmosphere (Figure S32, Supporting Information). Additionally, no hydrazine (N_2_H_4_) by‐product is found for NRR at −0.5 V versus RHE, revealing the excellent selectivity for electrosynthesis of NH_3_ with COF‐Fe/MXene‐F_17_ (Figures S33–S35, Supporting Information).^[^ ^31^ ^]^

**Figure 4:**
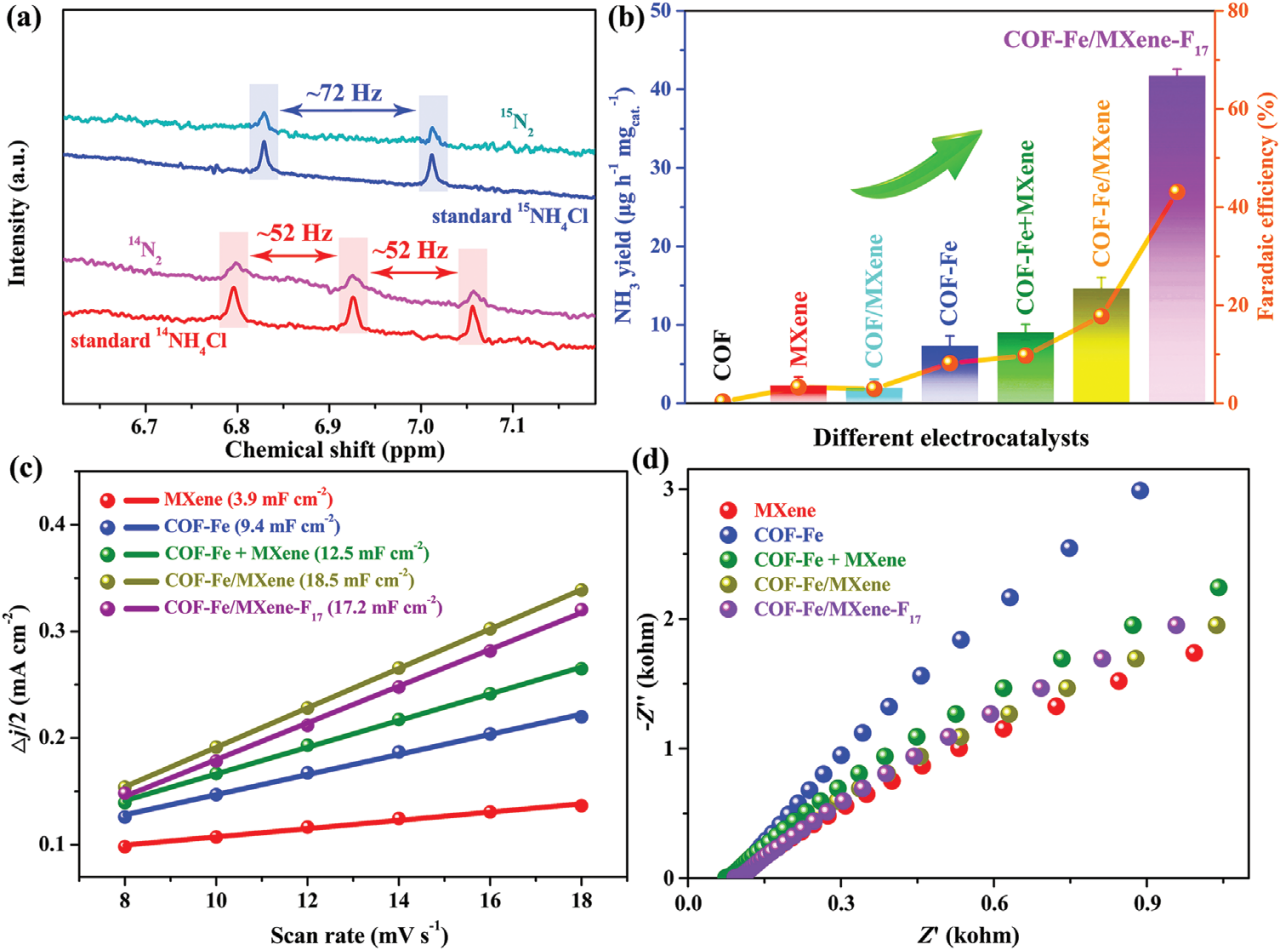
a) ^1^H NMR spectra of standard NH_4_Cl samples and post‐electrolytes using ^15^N_2_ and ^14^N_2_. b) The NRR performances of different catalysts. c) The relationship between the scan rate and plot of current density difference (Δ*j*/2) at 0.39 V versus RHE. d) Nyquist plots of MXene, COF‐Fe, COF‐Fe + MXene, COF‐Fe/MXene, and COF‐Fe/MXene‐F_17_.

Moreover, the ^15^N_2_ isotopic labeling NRR was explored for NH_3_ synthesis in the coexistence of COF‐Fe/MXene‐F_17_ and N_2_ at −0.5 V versus RHE. Due to the distinctly different ^1^H NMR spectra of ^14^NH_4_Cl and ^15^NH_4_Cl, the post‐electrolytes using ^14^N_2_ and ^15^N_2_ as feeding gases were analyzed by ^1^H NMR spectroscopy (**Figure** **4**a). The ^1^H NMR spectra of standard ^14^NH_4_Cl and ^15^NH_4_Cl samples were used as references. The ^1^H NMR spectrum for NH_3_ product in saturated ^14^N_2_ atmosphere shows triplet peaks with a coupling constant of ≈52 Hz as that of the standard ^14^NH_4_Cl sample. The post‐electrolyte of COF‐Fe/MXene‐F_17_ in ^15^N_2_ at −0.5 V versus RHE and commercial ^15^NH_4_Cl have two separate peaks in the ^1^H NMR spectra with a coupling constant of ≈72 Hz. Moreover, the production yields of ^14^N‐ labeled and ^15^N‐labeled NH_3_ quantified by ^1^H NMR are similar (Figure 3e). These results unequivocally suggest that the N_2_ gas is the only nitrogen source for electrocatalytic NH_3_ synthesis.

A series of control NRR tests using different electrocatalysts were measured at −0.5 V versus RHE in N_2_ condition to explore the reason for the vastly improved electrocatalytic performance of COF‐Fe/MXene‐F_17_ (Figure 4b and Figures S36 and S37, Supporting Information). The MXene, COF, and COF/MXene exhibit negligible electrochemical activities, but the COF‐Fe and COF‐Fe/MXene clearly show electrocatalytic NRR properties, which confirm the leading Fe catalytic sites for NRR via the interaction between Fe 3d and N 2p orbitals.^[^ ^32^ ^]^ The PXRD patterns suggest the similar crystalline phases of COF‐Fe and pristine COF (Figure S38, Supporting Information). The full XPS spectrum and high‐resolution Fe 2p XPS spectrum of COF‐Fe (Figures S39 and S40, Supporting Information) confirm the existence of Fe, with the maximum Fe content of ≈7.1 wt% as revealed by ICP‐OES. Meanwhile, COF‐Fe and COF‐Fe/MXene exhibit the similar XPS spectra of C 1s, N 1s, and Fe 2p, illustrating that both samples have the same oxidative state of relevant elements (Figure 1b and Figures S40 and S41, Supporting Information). The NRR performance of COF‐Fe/MXene is significantly better than that of COF‐Fe owing to the synergistic effect of COF‐Fe and MXene. The electrochemical active surface area can be evaluated by using double‐layer capacitance (*C*_dl_). The *C*_dl_ values of MXene and COF‐Fe were calculated to be 3.9 and 9.4 mF cm^−2^. In contrast, the COF‐Fe/MXene hybrid possesses a considerably increasing *C*_dl_ value of 18.5 mF cm^−2^, which indicates that the MXene nanosheets can not only support the COF‐Fe layers but also make the electrolyte easily accessible to the active catalytic surfaces (Figure 4c and Figures S42–S44, Supporting Information).^[^ ^33^ ^]^ Also, the Nyquist plots of COF‐Fe/MXene show a smaller electron transport resistance than COF‐Fe, which reveals the improved electron transfer kinetic after introducing MXene (Figure 4d). The synergistic effect in COF‐Fe/MXene can be further confirmed by the electrocatalytic NRR measurement of a physical mixture (COF‐Fe + MXene) of MXene (≈62 wt%) and COF‐Fe (≈38 wt%) containing the same Fe content as COF‐Fe/MXene (Figure 4b). Compared with COF‐Fe/MXene (18.5 mF cm^−2^), this mixture of COF‐Fe and MXene possesses a lower *C*_dl_ value of 12.5 mF cm^−2^ and a more enormous electron transport resistance due to the inhomogeneity and weaker interactions between the two species (Figure 4c,d and Figure S45, Supporting Information).^[^ ^34^ ^]^ This reveals that the in situ synthetic approach is beneficial for constructing the homogeneous and high‐efficiency COF‐based hybrid electrocatalysts.

**Figure 5:**
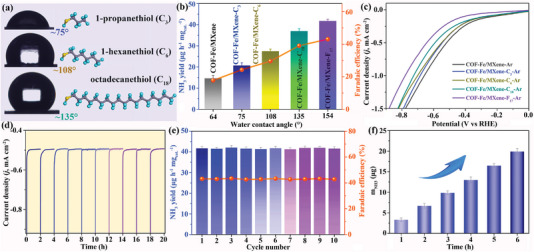
a) Water contact angle images of COF‐Fe‐MXene‐C_3_, COF‐Fe‐MXene‐C_6_, and COF‐Fe‐MXene‐C_18_. b) NRR performances and c) LSV curves under the saturated Ar atmosphere of different materials. d) CA curves and e) electrocatalytic NRR performances of COF‐Fe/MXene‐F_17_ for consecutive ten recycles. f) The amount of produced NH_3_ with different durations.

More importantly, COF‐Fe/MXene‐F_17_ exhibits 2.9‐ and 2.4‐folds increase in NH_3_ yield and Faradaic efficiency, respectively, compared with that of COF‐Fe/MXene. In fact, the *C*_dl_ value and Nyquist plots of COF‐Fe/MXene‐F_17_ are similar to those of COF‐Fe/MXene (Figure 4c,d and Figure S46, Supporting Information), but COF‐Fe/MXene‐F_17_ shows a greatly larger static water contact angle than that of COF‐Fe/MXene (Figure 1d). The enhanced NRR performance thus is mainly attributed to the introduction of hydrophobic layer. Furthermore, the hydrophobic behavior of COF‐Fe/MXene can be well controlled by regulating the alkyl molecules, including 1‐propanethiol (C_3_), 1‐hexanethiol (C_6_), and octadecanethiol (C_18_). All these hydrophobic COF‐Fe/MXene composites possess similar nanosheet morphologies and structures to that of COF‐Fe/MXene as revealed by TEM images and FT‐IR (Figures S47–S50, Supporting Information). The water contact angle measurements indicate that the hydrophobicity of composites increases with the total length of alkyl and fluoroalkyl chains, COF‐Fe/MXene (≈64°) < COF‐Fe/MXene‐C_3_ (≈75°) < COF‐Fe/MXene‐C_6_ (≈108°) < COF‐Fe/MXene‐C_18_ (≈135°) < COF‐Fe/MXene‐F_17_ (≈154°), because the longer alkyl and fluoroalkyl chains can repel the water more easily (Figure 1d and **Figure** **5**a). To further illustrate the relationship between hydrophobicity and N_2_‐to‐NH_3_ performance, these electrocatalysts were applied at −0.5 V versus RHE under the N_2_ atmosphere. The catalytic activity will be enhanced as the hydrophobicity increases (Figure 5b, Figures S51 and S52, Supporting Information). Also, the current density in the Ar‐saturated environment is COF‐Fe/MXene > COF‐Fe/MXene‐C_3_ > COF‐Fe/MXene‐C_6_ > COF‐Fe/MXene‐C_18_ > COF‐Fe /MXene‐F_17_ (Figure 5c). That is, the increasing alkyl length will boost the hydrophobicity and thus, suppress the HER to accelerate the NRR.^[^ ^35^ ^]^ Moreover, the hydrophobic effect of electrocatalysts can be verified by a physical mixture (PC/COF‐Fe/MXene) of COF‐Fe/MXene (50 wt%) and porous carbon (PC, 50 wt%). Compared with COF‐Fe/MXene, the hydrophobic PC/COF‐Fe/MXene shows more excellent NRR performance (Figures S53–S55, Supporting Information). The effective HER inhibition of PC/COF‐Fe/MXene was confirmed by the water contact angle and LSV curves under Ar atmosphere (Figures S56 and S57, Supporting Information).

The electrocatalytic stability of COF‐Fe/MXene‐F_17_ was measured by ten recycles at −0.5 V versus RHE for 2 h. The Faradaic efficiency and NH_3_ yield keep well in cyclic tests with similar CA curves, revealing the good stability of COF‐Fe/MXene‐F_17_ during the NRR process (Figure 5d,e). Moreover, the NH_3_ production amount increases along with the reaction time (Figure 5f and Figure S58, Supporting Information), which shows that NH_3_ is indeed generated from NRR catalyzed by using COF‐Fe/MXene‐F_17_. The COF‐Fe/MXene‐F_17_ after NRR was characterized by TEM, XPS, FT‐IR, HAADF‐STEM, and EDS mappings, which further reveal the nanosheet morphology of the used electrocatalyst with highly dispersed Fe active sites and 1H,1H,2H,2H‐perfluorodecanethiol (Figures S59–S62, Supporting Information).

**Figure 6:**
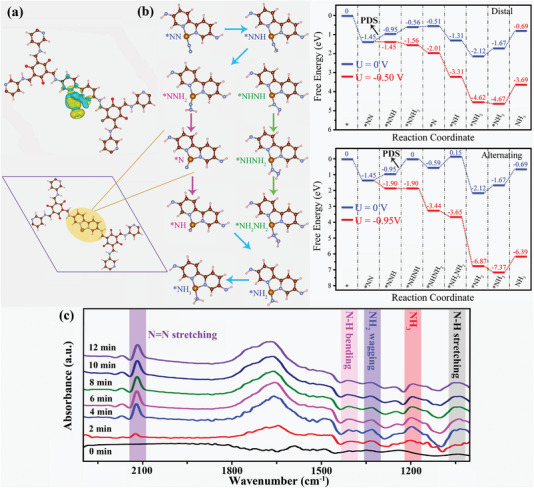
DFT calculations: a) The charge density of optimized N_2_‐adsorbed configuration on COF‐Fe. b) The free‐energy diagram and geometric structures of intermediates during the NRR process. c) Time‐resolved in situ electrochemical FT‐IR spectra on COF‐Fe/MXene‐F_17_.

Density functional theory (DFT) simulations were taken to study the N_2_‐to‐NH_3_ mechanism on COF‐Fe. The result shows that the end‐on coordination mode is more energetically favorable for N_2_ adsorption on the Fe sites (**Figure** **6**a). Two different kinetic pathways, that is, distal and alternating pathways, for N_2_ reduction are considered. The free‐energy diagrams and geometric structures of intermediates were explored by the DFT calculations. The optimal distal pathway is N_2_ → *NN → *NNH → *NNH_2_ → *N → *NH → *NH_2_ → *NH_3_ → NH_3_, while the optimal alternating pathway is N_2_ → *NN → *NNH → *NHNH → *NHNH_2_ → *NH_2_NH_2_ → *NH_2_ → *NH_3_ → NH_3_. Considering the superhigh bond energy of N≡N, the theoretical rate‐limiting step should be from NN* to NNH*. Both pathways have the same free energy barrier of 0.5 eV for hydrogenation reduction of *NN to *NNH intermediate, which is the potential‐determining step (PDS) in the distal pathway. However, the successive hydrogenation of *NNH → *NHNH with a maximum free energy barrier of 0.95 eV is considered as the PDS in the alternating path, which is higher than the PDS (0.5 eV) in the distal way. Hence, the distal path is more prone to happen during the N_2_ reduction (Figure 6b).

The time‐resolved in situ electrochemical FT‐IR spectra were recorded at −0.5 V versus RHE under the N_2_ atmosphere in 0.1 m Na_2_SO_4_ solution to explore the actual activation mechanism of COF‐Fe/MXene‐F_17_ toward N_2_ (Figure 6c). The FT‐IR peaks for the adsorbed NH_3_ (*NH_3_) at ≈1198 cm^−1^, N=N stretching at ≈2118 cm^−1^, and N—N stretching at ≈1041 cm^−1^ are found. In addition, all peak intensities gradually enhance with the increased time, which further confirms that the NH_3_ product originates from the electroreduction of N_2_ molecules.^[^ ^36^ ^]^ Some absorption peaks of —N*_x_* H*_y_* intermediates such as —N—H bending (≈1410 cm^−1^) and —NH_2_ wagging vibration (≈1328 cm^−1^) are also observed during the electroreduction process.^[^ ^36^ ^]^ These results evidently confirm that the N_2_ molecules can be efficiently activated by COF‐Fe/MXene‐F_17_.

Further, the Fe catalytic sites are replaced by other metals to synthesize COF‐Cu/MXene‐F_17_, COF‐Co/MXene‐F_17_, and COF‐Ni/MXene‐F_17_ for electrocatalytic NRR toward NH_3_ synthesis. The structures and compositions of these three hybrid materials were explored by FT‐IR, XPS, HAADF‐STEM, and EDS mappings (Figures S63–S69, Supporting Information). As similarly observed in COF‐Fe/MXene‐F_17_, 1H,1H,2H,2H‐perfluorodecanethiol, and metal sites could be successfully introduced in the customized hybrids. The F species mass fractions are ≈3.0, ≈3.1, and ≈2.9 wt% in COF‐Cu/MXene‐F_17_, COF‐Co/MXene‐F_17_, and COF‐Ni/MXene‐F_17_, and the Cu^2+^, Co^2+^, and Ni^2+^ in the corresponding hydrophobic electrocatalysts are ≈2.6, ≈2.4, and ≈2.5 wt%, respectively, as determined by ICP‐OES and EDS. The NH_3_ yields, at −0.5 V versus RHE in saturated N_2_ condition, are 18.94 µg h^−1^ mg_cat._^−1^ for COF‐Cu/MXene‐F_17_, 24.86 µg h^−1^ mg_cat._^−1^ for COF‐Ni/MXene‐F_17_, and 30.78 µg h^−1^ mg_cat._^−1^ for COF‐Co/MXene‐F_17_, with the Faradaic efficiencies of 21.3%, 26.9%, and 33.1%, respectively, which are NRRs effectively while clearly lower than that of COF‐Fe/MXene‐F_17_ (Figures S70–S72, Supporting Information). The results undoubtedly reveal the generality and feasibility of 2D metallized COF/MXene nanosheets for N_2_ electroreduction toward NH_3_ in aqueous solution under mild conditions.

## 3 Conclusion

In summary, a series of COF‐Fe/MXene nanosheets with adjustable hydrophobicity have been successfully designed and synthesized, the electrocatalytic NRR performances of which will be gradually enhanced with the increasing hydrophobicity. The COF‐Fe/MXene‐F_17_ with the most excellent hydrophobicity shows the maximum NH_3_ yield of 41.8 µg h^−1^ mg_cat._^−1^ and the highest Faradaic efficiency of 43.1% at −0.5 V versus RHE under ambient conditions. This work offers an operative strategy to design of high‐efficiency non‐noble metal NRR electrocatalysts.

## 4 Experimental Section

### Synthesis of COF/MXene Nanosheet

Tp (20 mg) was dissolved into CH_2_Cl_2_ (100 mL) in a beaker and water (60 mL) was added as an interlayer of the reaction system. Bpy (20.8 mg) was dissolved in a mixture of MeCN (30 mL) and water (70 mL), and amino‐functionalized Ti_3_C_2_T*_x_* MXene (30 mg) was ultrasonically dispersed in water (20 mL), which were then added into the Tp solution and maintained at room temperature for 3 days. The COF/MXene nanosheets were formed and collected after washing with water, DMF, acetone, and THF, respectively.

### Synthesis of COF‐Fe/MXene Nanosheet

COF/MXene (100 mg) was ultrasonically dispersed in MeOH (50 mL) for 30 min. Different amounts of FeCl_3_ (20, 30, and 40 mg) were dissolved in MeOH (20 mL). Both solutions were mixed and stirred at room temperature for 1 day. The COF‐Fe/MXene nanosheet was obtained after filtering and washing with fresh MeOH and HCl solution (0.1 m). The Fe contents in COF‐Fe/MXene nanosheets were determined as ≈1.6, ≈2.4, and ≈2.5 wt% by ICP‐OES.

### Synthesis of Hydrophobic COF‐Fe/MXene Nanosheet

COF‐Fe/MXene (30 mg) was added to a 10 mL EtOH solution of 4‐aminostyrene (119 mg), which was stirred at room temperature for 1 day. The product (20 mg) was added into a benzotrifluoride (2.7 mL) solution with excess hydrophobic component (1 mmol), such as 1‐propanethiol, 1‐hexanethiol, octadecanethiol, and 1H,1H,2H,2H‐perfluorodecanethiol. After that, azobisisobutyronitrile (5 mg) was added, which was heated at 333 K in N_2_ atmosphere for 3 h. The hydrophobic COF‐Fe/MXene nanosheet was filtered and stirred in ethanol for 1 day.

Other hydrophobic metallized COF/MXene nanosheets, such as COF‐Cu/MXene‐F_17_, COF‐Co/MXene‐F_17_, and COF‐Ni/MXene‐F_17_, were similarly prepared using the synthetic procedure of COF‐Fe/MXene‐F_17_. In these cases, COF/MXene (100 mg) was immersed into the methanol solutions (20 mL) of CuCl_2_ (40 mg), CoCl_2_ (40 mg), and NiCl_2_ (40 mg), respectively.

### Time‐Resolved In Situ Electrochemical FT‐IR Spectra

Time‐resolved in situ electrochemical FT‐IR spectra were recorded in a 0.1 m Na_2_SO_4_ aqueous solution. The glassy carbon electrode (GCE) was modified by COF‐Fe/MXene‐F_17_, using a saturated calomel electrode (SCE) and a Pt plate as the reference and counter electrodes, respectively. The electrochemical FT‐IR spectra were obtained after aerating nitrogen gas to the spectro‐electrochemical cell with electrolyte for 30 min. The background FT‐IR spectrum was taken, then the FT‐IR spectra were collected with this background at −0.5 V versus RHE under the N_2_‐saturated condition.

### Preparation of Working Electrode

Electrocatalyst (4 mg) was well dispersed in a mixture of Nafion (50 µL) and isopropanol (950 µL), which was ultrasonicated to achieve a homogeneous ink. The prepared ink (20 µL) was slowly added on the surface of GCE, producing the working electrode after drying in air.

### Electrochemical Measurements

Ar and N_2_ gases were passed through the KOH and H_2_SO_4_ water solutions (0.1 m) to remove the possible N‐containing contaminant. The electrocatalytic process was taken in a H‐type two‐compartment cell with commercial N117 Nafion membrane. The Nafion membrane was pretreated by soaking into a H_2_O_2_ (5 wt%) aqueous solution for 1 h at 80 °C, which was further immersed in deionized water for another 0.5 h. Then, the membrane was soaked into a H_2_SO_4_ (5 wt%) solution for 1 h at 353 K and the deionized water for 0.5 h, respectively. Electrochemical measurements were performed on an electrochemical station in a 0.1 m Na_2_SO_4_ water solution (30 mL) at room temperature. Catalysts were used to modify the GCE working electrode with a SCE as reference electrode and a Pt plate as counter electrode. The electrolyte was treated through three freeze‐pumping processes to remove the residual air. Prior to each NRR test, the N_2_ gas was continuously flowed through the catalytic system for 30 min. The NRR process was taken at different applied potentials in N_2_‐saturated condition for 2 h. The applied potential was converted to RHE via *E*_(RHE)_ = *E*_(SCE)_ + 0.0591 × pH + 0.242 V.

### Computational Details

All spin‐polarized calculations were performed with Vienna ab initio simulation package.^[^ ^37^, ^38^ ^]^ The ion–electron interaction was described using the projector augmented wave method.^[^ ^39^ ^]^ The general gradient approximation in Perdew–Burke–Ernzerhof form was used.^[^ ^40^, ^41^ ^]^ Herein, Grimme's semiempirical DFT‐D3 method was used to treat the weak van der Waals interactions between adsorbate and substrate.^[^ ^42^ ^]^ The cut‐off energy for the plane‐wave basis was set to 500 eV. The Γ‐centered k‐point meshes of 2 × 2 × 1 and 3 × 3 × 1 based on the Monkhorst–Pack scheme were used for the geometric and electronic structure calculations, respectively.^[^ ^43^ ^]^ During the structure relaxation, the convergence criterion was set to 0.02 eV Å^−1^ and 10^−5^ eV for residual force and total energy, respectively and in order to avoid the interactions between two periodic units, a vacuum space of 20 Å was adopted.

The free energy change (Δ*G*) of each elementary reaction was calculated as

(1) $$\Delta G=\Delta E+\Delta E_{\mathrm{ZPE}}-T\Delta S$$

in which Δ*E*, *E*_ZPE_, *T*, and *S* are the reaction energy difference, zero‐point energies, temperature, and entropy, respectively.

## Conflict of Interest

The authors declare no conflict of interest.

## Supporting information

Supporting Information

## Data Availability

The data that support the findings of this study are available from the corresponding author upon reasonable request.

## References

[1] a) J. Lim , C. A. Fernández , S. W. Lee , M. C. Hatzell , ACS Energy Lett. 2021, 6, 3676;

[2] C. Smith , A. K. Hill , L. Torrente‐Murciano , Energy Environ. Sci. 2020, 13, 331.

[3] a) J. G. Chen , R. M. Crooks , L. C. Seefeldt , K. L. Bren , R. M. Bullock , M. Y. Darensbourg , P. L. Holland , B. Hoffman , M. J. Janik , A. K. Jones , M. G. Kanatzidis , P. King , K. M. Lancaster , S. V. Lymar , P. Pfromm , W. F. Schneider , R. R. Schrock , Science 2018, 360, eaar6611; 29798857 10.1126/science.aar6611 PMC6088796

[4] Y. Zhang , Q. Zhang , D.‐X. Liu , Z. Wen , J.‐X. Yao , M.‐M. Shi , Y.‐F. Zhu , J.‐M. Yan , Q. Jiang , Appl. Catal., B 2021, 298, 120592.

[5] H. Zhong , M. Wang , M. Ghorbani‐Asl , J. Zhang , K. H. Ly , Z. Liao , G. Chen , Y. Wei , B. P. Biswal , E. Zschech , I. M. Weidinger , A. V. Krasheninnikov , R. Dong , X. Feng , J. Am. Chem. Soc. 2021, 143, 19992. 34784212 10.1021/jacs.1c11158

[6] G. A. Cerron‐Calle , A. S. Fajardo , C. M. Sanchez‐Sanchez , S. Garcia‐Segura , Appl. Catal., B 2022, 302, 120844.

[7] C. Ma , Y. Zhang , S. Yan , B. Liu , Appl. Catal., B 2022, 315, 121574.

[8] a) C. J. M. van der Ham , M. T. M. Koper , D. G. H. Hetterscheid , Chem. Soc. Rev. 2014, 43, 5183; 24802308 10.1039/c4cs00085d

[9] S.‐Y. Ding , W. Wang , Chem. Soc. Rev. 2013, 42, 548. 23060270 10.1039/c2cs35072f

[10] a) V. Hasija , S. Patial , P. Raizada , A. A. P. Khan , A. M. Asiri , Q. V. Le , V.‐H. Nguyen , P. Singh , Coord. Chem. Rev. 2022, 452, 214298;

[11] a) Y. Liu , W. Li , C. Yuan , L. Jia , Y. Liu , A. Huang , Y. Cui , Angew. Chem., Int. Ed. 2022, 61, e202113348; 10.1002/anie.202113348 34710948

[12] a) Y. Bai , Y. Liu , M. Liu , X. Wang , S. Shang , W. Gao , C. Du , Y. Qiao , J. Chen , J. Dong , Y. Liu , Angew. Chem., Int. Ed. 2022, 61, e202113067; 10.1002/anie.202113067 34699115

[13] M. Naguib , V. N. Mochalin , M. W. Barsoum , Y. Gogotsi , Adv. Mater. 2014, 26, 992. 24357390 10.1002/adma.201304138

[14] a) N. Liu , L. Yu , B. Liu , F. Yu , L. Li , Y. Xiao , J. Yang , J. Ma , Adv. Sci. 2023, 10, 2204041; 10.1002/advs.202204041 PMC9839853 36442852

[15] a) D. Guo , F. Ming , D. B. Shinde , L. Cao , G. Huang , C. Li , Z. Li , Y. Yuan , M. N. Hedhili , H. N. Alshareef , Z. Lai , Adv. Funct. Mater. 2021, 31, 2101194;

[16] a) D. Rodriguez‐San‐Miguel , C. Montoro , F. Zamora , Chem. Soc. Rev. 2020, 49, 2291; 32182308 10.1039/c9cs00890j

[17] a) B. Hinnemann , J. K. Nørskov , J. Am. Chem. Soc. 2003, 125, 1466; 12568592 10.1021/ja029041g

[18] Q. Sun , B. Aguila , J. A. Perman , T. Butts , F.‐S. Xiao , S. Ma , Chem 2018, 4, 1726.

[19] K. Dey , M. Pal , K. C. Rout , S. Kunjattu H , A. Das , R. Mukherjee , U. K. Kharul , R. Banerjee , J. Am. Chem. Soc. 2017, 139, 13083. 28876060 10.1021/jacs.7b06640

[20] B. Ma , H. Zhao , T. Li , Q. Liu , Y. Luo , C. Li , S. Lu , A. M. Asiri , D. Ma , X. Sun , Nano Res. 2021, 14, 555.

[21] a) G. Zhang , T. Wang , Z. Xu , M. Liu , C. Shen , Q. Meng , Chem. Commun. 2020, 56, 11283; 10.1039/d0cc04265j 32839809

[22] Q. Pan , M. Abdellah , Y. Cao , W. Lin , Y. Liu , J. Meng , Q. Zhou , Q. Zhao , X. Yan , Z. Li , H. Cui , H. Cao , W. Fang , D. A. Tanner , M. Abdel‐Hafiez , Y. Zhou , T. Pullerits , S. E. Canton , H. Xu , K. Zheng , Nat. Commun. 2022, 13, 845. 35149679 10.1038/s41467-022-28409-2 PMC8837612

[23] a) R. Bu , L. Zhang , L.‐L. Gao , W.‐J. Sun , S.‐L. Yang , E.‐Q. Gao , Mol. Catal. 2021, 499, 111319;

[24] a) M. Cai , S. Ding , B. Gibbons , X. Yang , M. C. Kessinger , A. J. Morris , Chem. Commun. 2020, 56, 14361; 10.1039/d0cc02206c 33140756

[25] a) X. Yao , C. Tang , G. Yuan , P. Cui , X. Xu , Z. Liu , Electrochem. Commun. 2011, 13, 1439;

[26] Q. Sun , H. He , W.‐Y. Gao , B. Aguila , L. Wojtas , Z. Dai , J. Li , Y.‐S. Chen , F.‐S. Xiao , S. Ma , Nat. Commun. 2016, 7, 13300. 27796363 10.1038/ncomms13300 PMC5095586

[27] a) D. B. Shinde , H. B. Aiyappa , M. Bhadra , B. P. Biswal , P. Wadge , S. Kandambeth , B. Garai , T. Kundu , S. Kurungot , R. Banerjee , J. Mater. Chem. A 2016, 4, 2682;

[28] a) Y. Zhao , R. Shi , X. Bian , C. Zhou , Y. Zhao , S. Zhang , F. Wu , G. I. N. Waterhouse , L.‐Z. Wu , C.‐H. Tung , T. Zhang , Adv. Sci. 2019, 6, 1802109; 10.1002/advs.201802109 PMC6468971 31016117

[29] A. C. Nielander , J. M. McEnaney , J. A. Schwalbe , J. G. Baker , S. J. Blair , L. Wang , J. G. Pelton , S. Z. Andersen , K. Enemark‐Rasmussen , V. Čolić , S. Yang , S. F. Bent , M. Cargnello , J. Kibsgaard , P. C. K. Vesborg , I. Chorkendorff , T. F. Jaramillo , ACS Catal. 2019, 9, 5797.

[30] a) X. Xiang , Z. Wang , X. Shi , M. Fan , X. Sun , ChemCatChem 2018, 10, 4530;

[31] W. Xu , G. Fan , J. Chen , J. Li , L. Zhang , S. Zhu , X. Su , F. Cheng , J. Chen , Angew. Chem., Int. Ed. 2020, 59, 3511. 10.1002/anie.201914335 31889387

[32] a) A. McSkimming , D. L. M. Suess , Nat. Chem. 2021, 13, 666; 34045715 10.1038/s41557-021-00701-6

[33] a) T. Ma , J. Cao , M. Jaroniec , S. Qiao , Angew. Chem., Int. Ed. 2016, 55, 1138; 10.1002/anie.201509758 26629779

[34] a) C.‐F. Du , K. N. Dinh , Q. Liang , Y. Zheng , Y. Luo , J. Zhang , Q. Yan , Adv. Energy Mater. 2018, 8, 1801127;

[35] a) H. He , Q.‐Q. Zhu , Y. Yan , H.‐W. Zhang , Z.‐Y. Han , H. Sun , J. Chen , C.‐P. Li , Z. Zhang , M. Du , Appl. Catal., B 2022, 302, 120840;

[36] a) Y. Yao , H. Wang , X.‐Z. Yuan , H. Li , M. Shao , ACS Energy Lett. 2019, 4, 1336;

[37] G. Kresse , J. Furthmüller , Phys. Rev. B 1996, 54, 11169. 10.1103/physrevb.54.11169 9984901

[38] G. Kresse , D. Joubert , Phys. Rev. B 1999, 59, 1758.

[39] P. E. Blöchl , Phys. Rev. B 1994, 50, 17953. 10.1103/physrevb.50.17953 9976227

[40] J. P. Perdew , J. Chevary , S. Vosko , K. A. Jackson , M. R. Pederson , D. Singh , C. Fiolhais , Phys. Rev. B 1992, 46, 6671. 10.1103/physrevb.46.6671 10002368

[41] J. P. Perdew , Y. Wang , Phys. Rev. B 1992, 45, 13244. 10.1103/physrevb.45.13244 10001404

[42] S. Grimme , J. Antony , S. Ehrlich , H. Krieg , J. Chem. Phys. 2010, 132, 154104. 20423165 10.1063/1.3382344

[43] H. J. Monkhorst , J. D. Pack , Phys. Rev. B 1976, 13, 5188.

