# Supplementary material for: Hydrophobicity Tailoring of Ferric Covalent Organic Framework/MXene Nanosheets for High‐Efficiency Nitrogen Electroreduction to Ammonia
Source: Adv Sci (Weinh). 2023 Mar 30;10(15):2206933. doi: 10.1002/advs.202206933 (PMC10214235; doi:10.1002/advs.202206933)
Supplement: Supplementary file 1 — Supporting Information [file ADVS-10-2206933-s001.pdf]

Supporting Information

Hydrophobicity Tailoring of Ferric Covalent Organic Framework/MXene Nanosheets for High-Efficiency Nitrogen Electroreduction to Ammonia

*Hongming He, Hao-Ming Wen, Hong-Kai Li, Ping Li, Jiajun Wang, Yijie Yang, Cheng-Peng Li, Zhihong Zhang and Miao Du**

Dr. H. He, H.-M. Wen, H.-K. Li, P. Li, Dr. J. Wang, Dr. Y. Yang, Prof. C.-P. Li
College of Chemistry, Tianjin Key Laboratory of Structure and Performance for Functional Molecules, Tianjin Normal University, Tianjin 300387, China

Prof. Z. Zhang, Prof. M. Du
College of Material and Chemical Engineering, Institute of New Energy Science and Technology, School of Future Hydrogen Energy Technology, Zhengzhou University of Light Industry, Zhengzhou 450001, China

E-mail: dumiao@zzuli.edu.cn

Section S1. Experimental Section

1. General materials and methods

Chemicals and reagents were purchased from commercial corporations. $^{14}N_2$ (≥ 99.999%) and Ar gases (≥ 99.999%) were obtained from Huanyu Co., Ltd. $^{15}N_2$ gas (99%) was obtained from Tianjin Taiya Co., Ltd. The 1,3,5-triformylphloroglucinol (Tp, >97%) and 2,2'-bipyridine-5,5'-diamine (Bpy, > 97%) were obtained from Shanghai Tensus Biotech Co., Ltd. Dichloromethane (CH_2Cl_2, AR), acetonitrile (MeCN, AR), *N*,*N*-dimethylformamide (DMF, AR), tetrahydrofuran (THF, AR), acetone (AR), methanol (MeOH, AR), ethanol (EtOH, AR), and hydrazine (N_2H_4, AR) were purchased from Tianjin Kermel Chemical Reagent Co., Ltd. Ferric chloride ($FeCl_3$, AR), 1-propanethiol (AR), 1-hexanethiol (AR), octadecanethiol (AR), sodium nitroferricyanide (AR), and $^{14}NH_4Cl$ (AR) were gained from Meryer (Shanghai)

Chemical Technology Co., Ltd. Hydrochloric acid (HCl, 36–38 wt.%) and nickel chloride ($NiCl_2$) were purchased from Tianjin Fengchuan Chemical Reagent Co., Ltd. $^{15}NH_4Cl$ (≥ 99%) was purchased from Sigma-Aldrich (Shanghai) Trading Co., Ltd. Amino-functionalized $Ti_3C_2T_x$ MXene (99.5%) was obtained from Beike 2D Materials Co., Ltd. 4-Aminostyrene (99.7%) and 1*H*,1*H*,2*H*,2*H*-perfluorodecanethiol (99.4%) were gained from Bide Pharmatech Ltd. Benzotrifluoride (99%) was gained from J&K Scientific. Azobisisobutyronitrile (99.8%), sodium sulfate (Na_2SO_4, 99.9%), deionized water, salicylic acid (99.7%), sodium citrate (99.5%), sodium hypochlorite (NaClO, 99.9%), and *para*-(dimethylamino) benzaldehyde (99.5%) were purchased from Shanghai Aladdin Biochemical Technology Co., Ltd. Cupric chloride ($CuCl_2$, AR) was purchased from Shanghai Yuanye Bio-Technology Co., Ltd. Cobalt chloride ($CoCl_2$, AR) was gained from Fuchen (Tianjin) Chemical Reagent Co., Ltd. Isopropanol (AR), KOH (AR), and H_2SO_4 acid (95–98%) were obtained from Tianjin Damao Chemical Reagent Co., Ltd. Hydrogen peroxide solution (35 wt.%) was gained from Alfa Aesar Chemical Co. Ltd. Potassium sodium tartrate (AR) and Nessler's reagent (AR) were purchased from Shanghai Macklin Biochemical Co., Ltd. Dimethyl sulfoxide (DMSO-d_6, 99.8%) was obtained from Energy Chemical.

Fourier transform infrared (FT-IR) spectra were taken on Bruker AlPHA FT-IR spectrometer. Time-resolved in situ electrochemical FT-IR spectra were performed on Bruker tensor II FT-IR spectrometer. Powder X-ray diffraction (PXRD) was taken on Rigaku Ultima IV diffractometer. Thermogravimetric analysis (TGA) was taken on Perkin-Elmer thermogravimetric analyzer in air at the heating rate of 10 °C min^{-1}. N_2 adsorption isotherm was taken on Micromeritics ASAP 2020 surface area analyzer at 77 K. Inductively coupled plasma optical emission spectrometry (ICP-OES) was taken on Thermo Scientific iCAP 6500 system. ^1H NMR spectra were taken on Bruker AV 400 NMR spectrometer (400 MHz). Transmission electron microscopy (TEM) and scanning electron microscopy (SEM) were

taken on Tecnai G^2 F20 and Nova Nano SEM 230 electron microscope. Atomic force microscopy (AFM) was performed on Bruker Multimode 8. microscopy. X-ray photoelectron spectroscopy (XPS) was taken on Thermo Fisher ESCALAB Xi+ spectrometer. Contact angles were measured on JY-82C apparatus. Ultraviolet-visible (UV-vis) spectra were taken on Mapada V-1200 spectrometer. Electrochemical tests were performed on CHI660D electrochemical workstation.

2. Synthesis

COF nanosheet. Tp (15.7 mg) was dissolved in CH_2Cl_2 (100 mL) in a 500 mL beaker, to which water (60 mL) was slowly added as the spacer-layer. Bpy (20.8 mg) was dissolved in a mixture of MeCN (30 mL) and water (70 mL), which was then added to the Tp solution and maintained at room temperature for 3 days. The COF nanosheets were afforded and collected after washing with water, DMF, acetone, and THF, respectively.[1]

COF-Fe nanosheet. The as-synthesized COF nanosheet (100 mg) was ultrasonically dispersed in MeOH (50 mL) for 30 min. Different amounts of $FeCl_3$ (20, 30, and 40 mg) were dissolved in MeOH (20 mL), which were then added to the methanol suspension of COF nanosheet. The reaction systems were stirred at room temperature for 1 day. The product was washed with fresh MeOH and HCl water solution (0.1 M). The Fe contents of different COF-Fe nanosheets were determined to be ~4.6, ~7.0, and ~7.1 wt% by ICP-OES.

3. Detection of ammonia

The synthesized ammonia was quantitatively determined by various approaches. Generally, the linear relationship of analytic signal and ammonia concentration was established accurately via a series of standard ammonia solutions to calculate the ammonia concentration in electrolyte.

Nessler's reagent approach.[2] 3 mL of post-electrolysis electrolyte was mixed with 0.3 mL of potassium sodium tartrate solution (0.2 M), which was then added with the Nessler's

reagent (0.3 mL). After the obtained solution was kept in the dark for 20 min, UV-vis absorption spectra were measured to analyze the intensity at 425 nm.

Indophenol blue method.[3] Solution A is a mixture of 1 M NaOH solution containing 5 wt% salicylic acid and 5 wt% sodium citrate. Solution B is a NaClO (0.2 M) water solution. Solution C is a 1 wt% sodium nitroferricyanide aqueous solution. The post-tested electrolyte solution (2 mL) was sequentially added with solution A (2 mL), solution B (1 mL) and solution C (0.2 mL). After keeping for 2 h at room temperature, the UV-vis absorption spectra of this mixed solution were recorded. The intensity of maximum absorbance at 655 nm was applied to determinate the ammonia yield.

^{1}H NMR spectra.[4] The ammonia solution (1 mL) was mixed with a H_2SO_4 water solution (1 mL, 0.1 M). Then, 0.5 mL of the mixed solution was added with DMSO-d_6 (0.05 mL), which was further analyzed by ^{1}H NMR spectra. The NRR test was measured under N_2 atmosphere at –0.5 V versus RHE for 4 h. The electrolyte was then concentrated to 3 mL for ^{1}H NMR test.

4. Detection of hydrazine

The Watt and Chrisp method was used for quantitative analysis of N_2H_4.[5] A mixed solution of *para*-(dimethylamino) benzaldehyde (4 g), concentrated HCl (20 mL), and EtOH (200 mL) was prepared as the color reagent. The hydrazine solution (5 mL) and the color reagent (5 mL) were mixed together for 15 min at room temperature and further measured the corresponding UV-vis absorption spectrum. The absorbance intensity at λ = 458 nm was employed to establish a linear equation with the concentration of hydrazine by diverse standard hydrazine concentrations. The UV-vis spectra of the electrolyte were collected to determine the hydrazine concentration.

5. Calculation of ammonia production rate and Faradaic efficiency

The Faradaic efficiency was calculated by the number of electric charges in ammonia synthesis divided by the total electric charges passing through the electrodes during

electrolysis:

$$\text{Faradaic efficiency} = \frac{3 \times F \times c_{NH_3} \times V}{Q} \times 100\%$$

The ammonia production rate (R_{NH_3}) is defined as the following equation:

$$R_{NH_3} = \frac{c_{NH_3} \times V}{t \times m_{cat.}}$$

in which F is the Faraday constant (96485 C mol^{-1}), c_{NH_3} is the ammonia concentration, V is the volume of electrolyte, Q is the total transferred charge during NRR, t is the electrochemical NRR time, and $m_{cat.}$ is the mass of catalyst.

6. Isotope labeling experiment

The ^{15}N isotopic labeled NRR experiment was taken with $^{15}N_2$ as the nitrogen source. The NRR test was conducted under $^{15}N_2$ atmosphere at –0.5 V versus RHE for 2 h and the electrolyte was concentrated to 3 mL. A concentrated electrolyte (1 mL) was mixed with H_2SO_4 water solution (1 mL, 0.1 M). Subsequently, 0.05 mL of DMSO-d_6 was added into the mixed solution (0.5 mL) for ^1H NMR measurement.

Section S2. Supplementary Figures

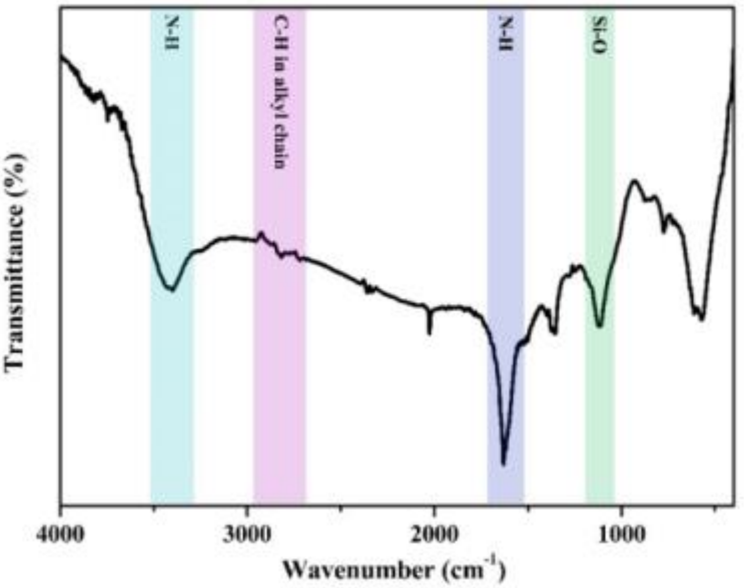

Figure S1. FT-IR spectrum of the alkylamino group modified MXene.

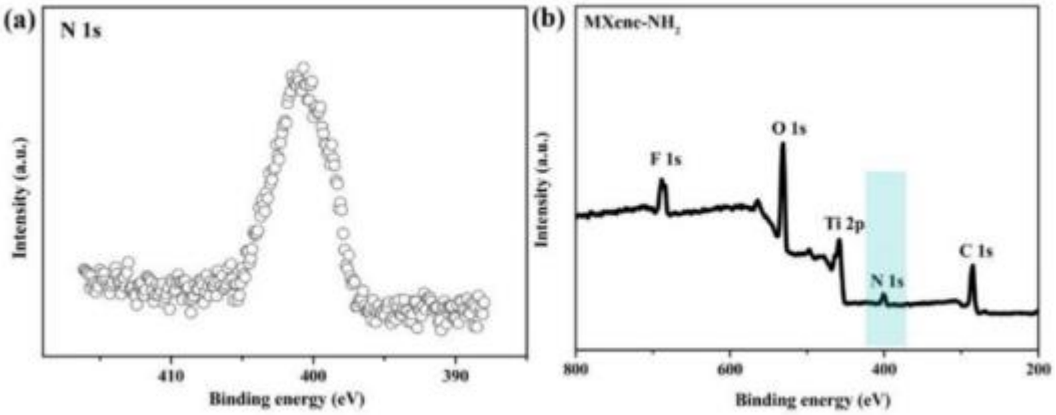

Figure S2. a) High-resolution N 1*s* XPS spectrum and b) full XPS spectrum of the alkylamino group modified MXene.

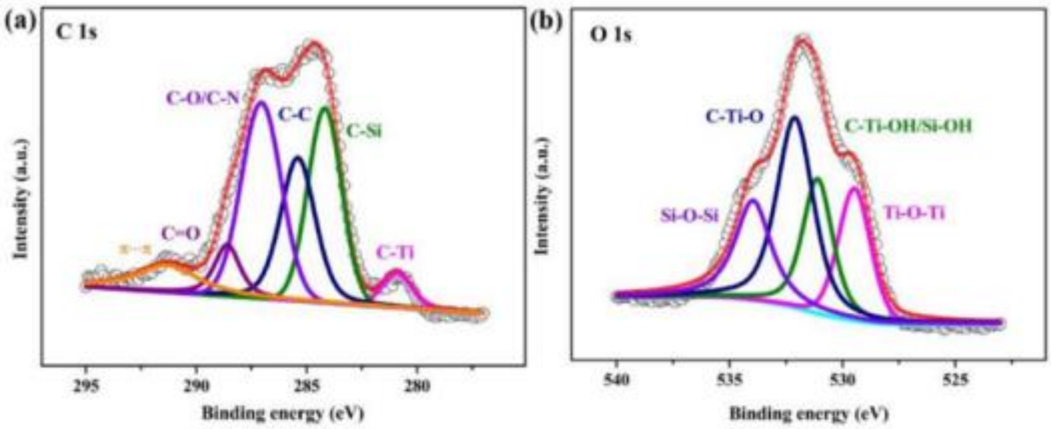

Figure S3. High-resolution XPS spectra of a) C 1*s* and b) O 1*s* of the alkylamino group modified MXene.

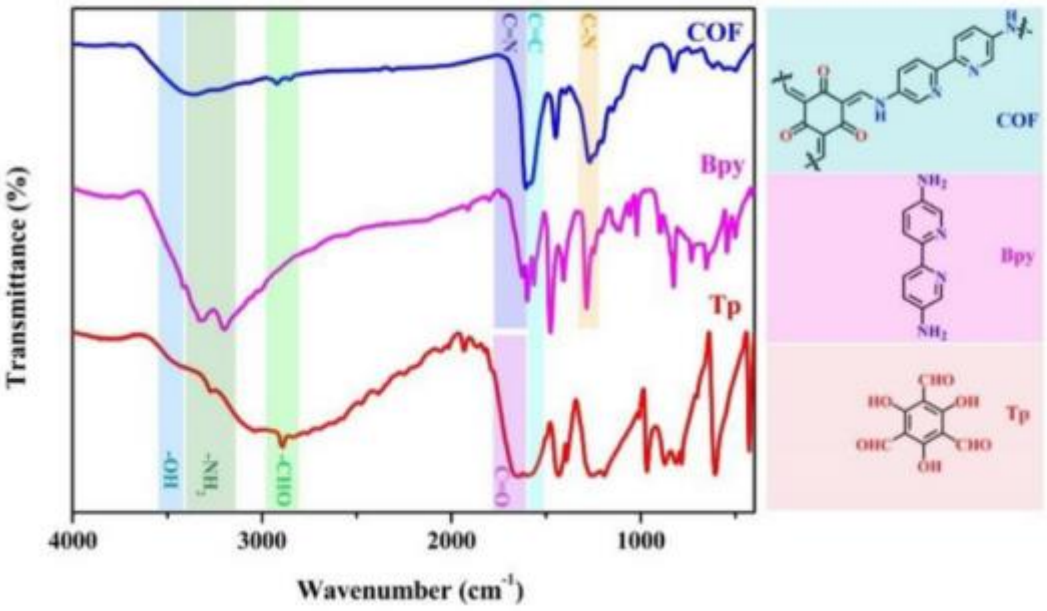

Figure S4. FT-IR spectra of COF and organic monomers.

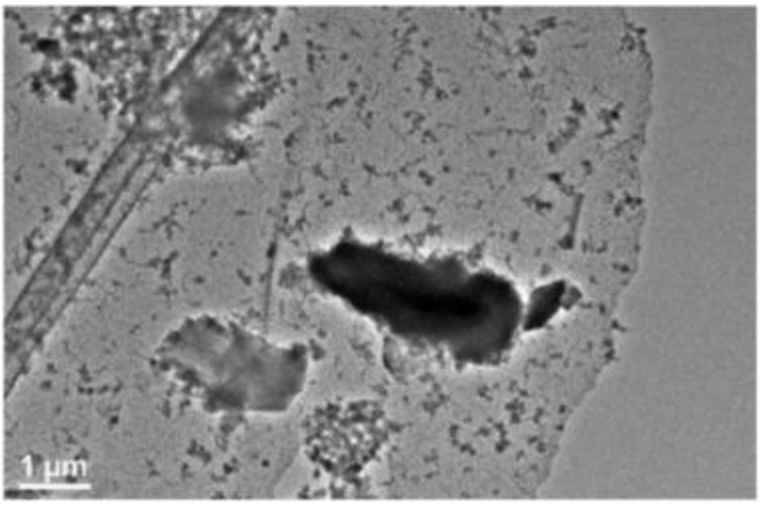

Figure S5. TEM image of COF/MXene prepared using the MXene without $–NH_2$.

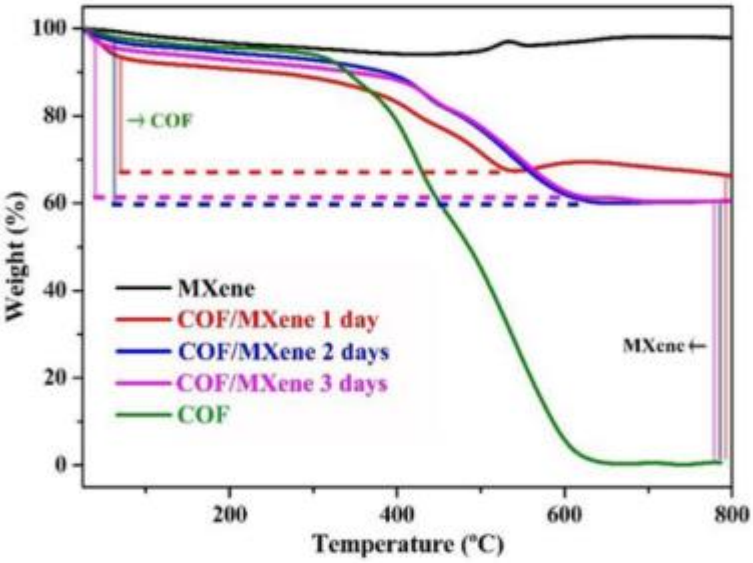

Figure S6. TGA curves of MXene, COF, and COF/MXene at different reaction days.

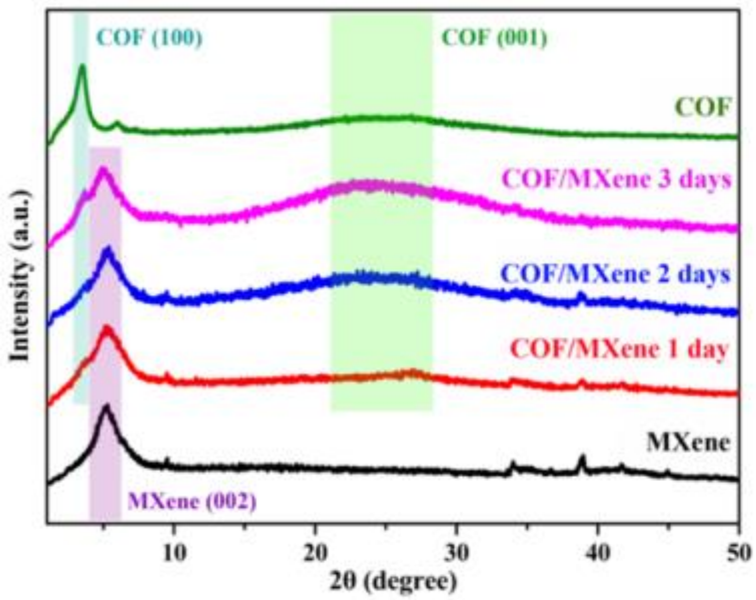

Figure S7. PXRD patterns of MXene, COF, and COF/MXene at different reaction days.

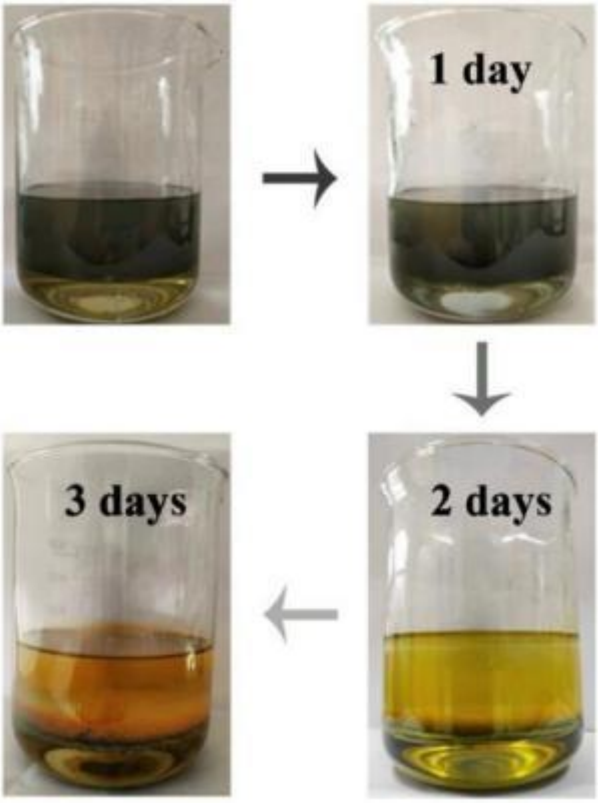

Figure S8. Optical images of COF/MXene at different reaction days.

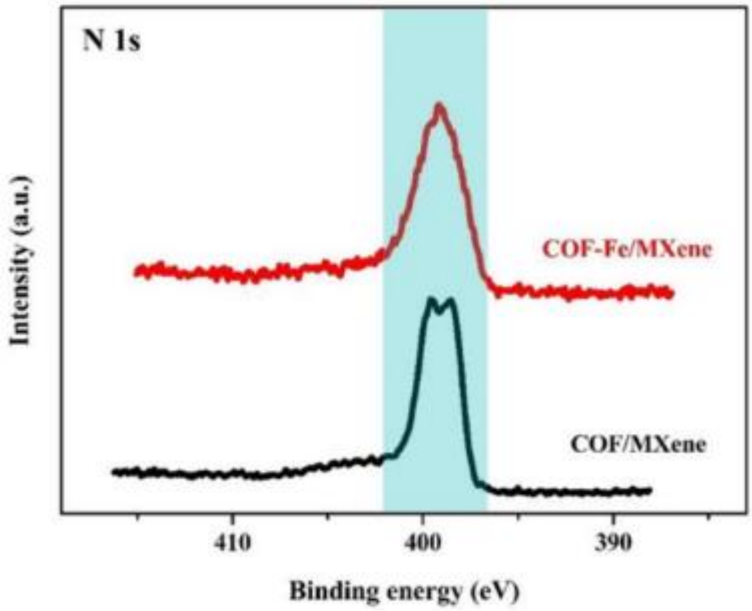

Figure S9. High-resolution N 1*s* XPS spectra of COF/MXene and COF-Fe/MXene.

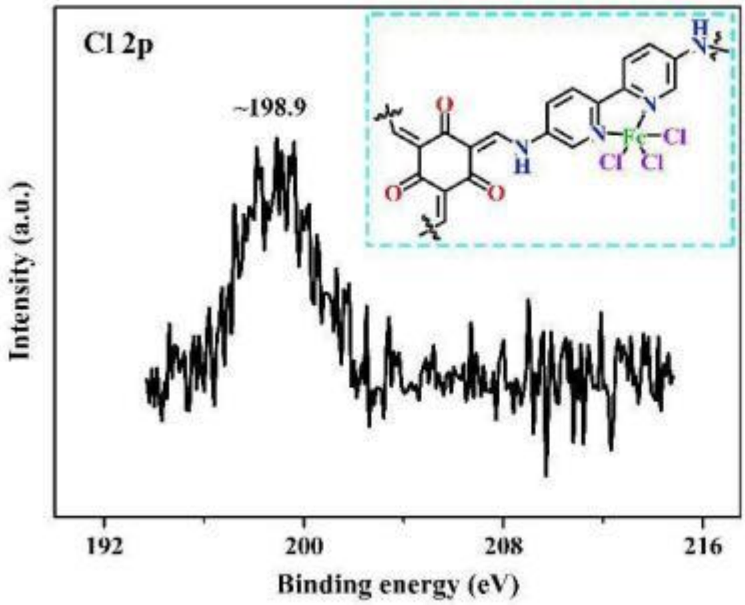

Figure S10. Cl 2*p* XPS spectrum of COF-Fe/MXene and the possible coordination environment.

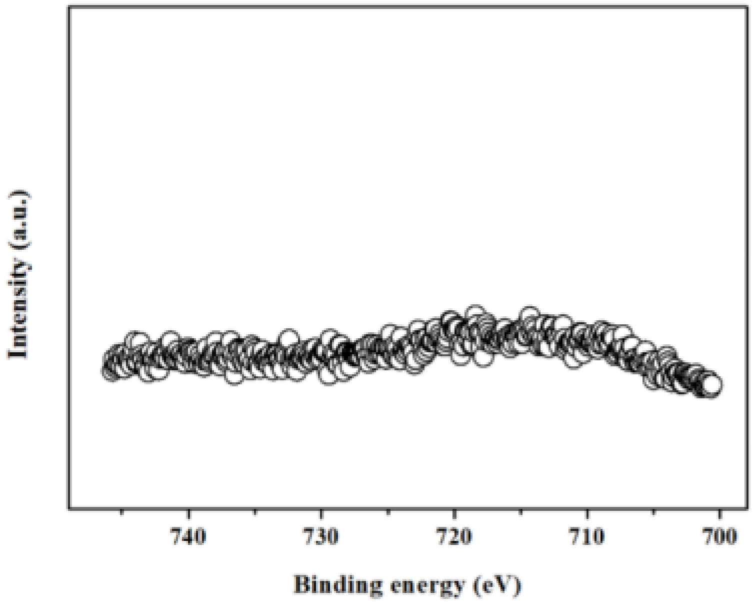

Figure S11. Fe 2*p* XPS signal of COF/MXene.

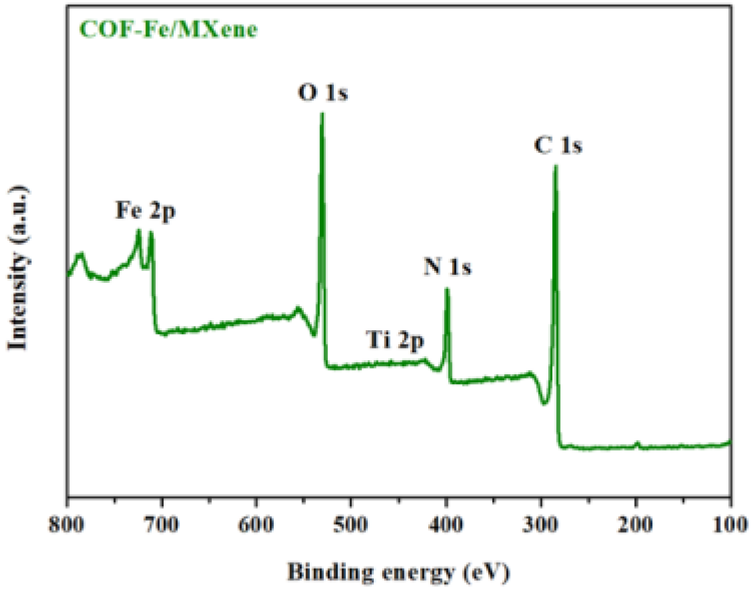

Figure S12. Full XPS spectrum of COF-Fe/MXene.

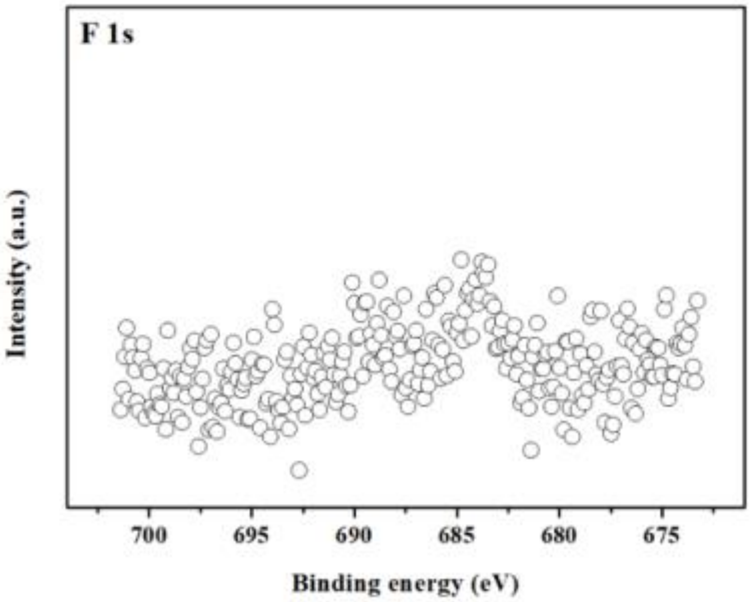

Figure S13. High-resolution F 1*s* XPS signal of COF-Fe/MXene.

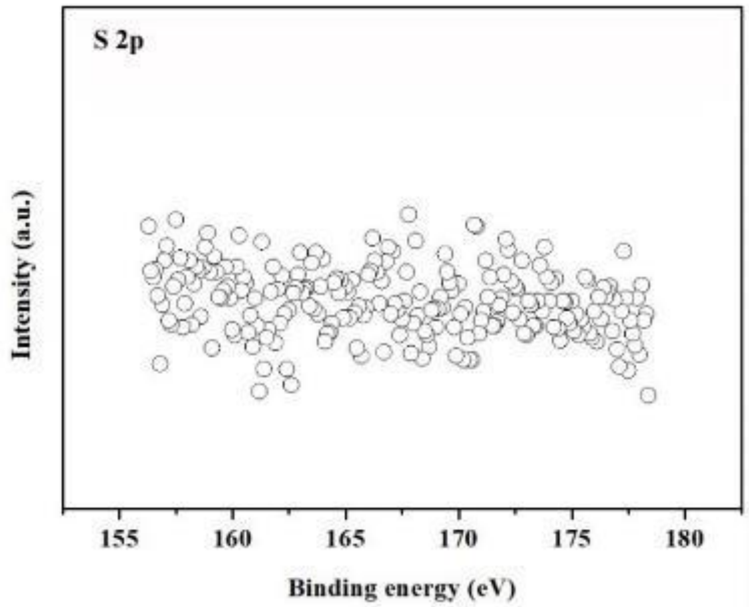

Figure S14. High-resolution S 2*p* XPS signal of COF-Fe/MXene.

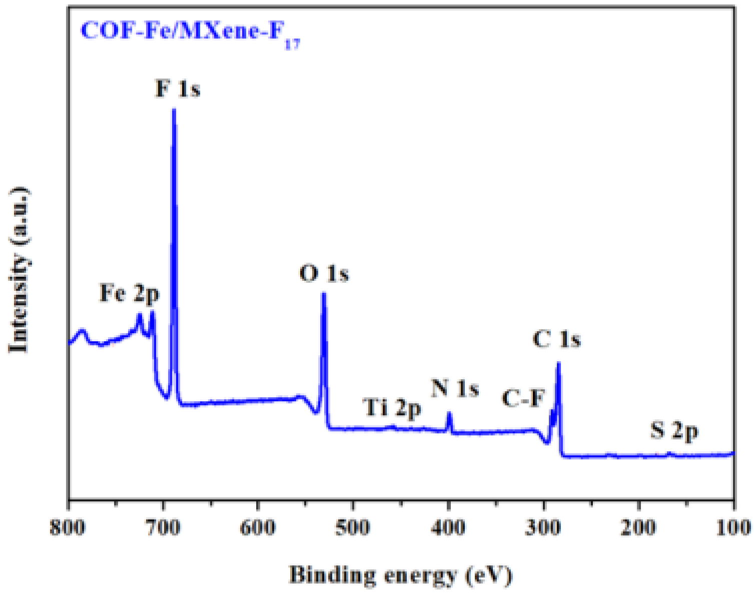

Figure S15. Full XPS spectrum of COF-Fe/MXene-F_{17}.

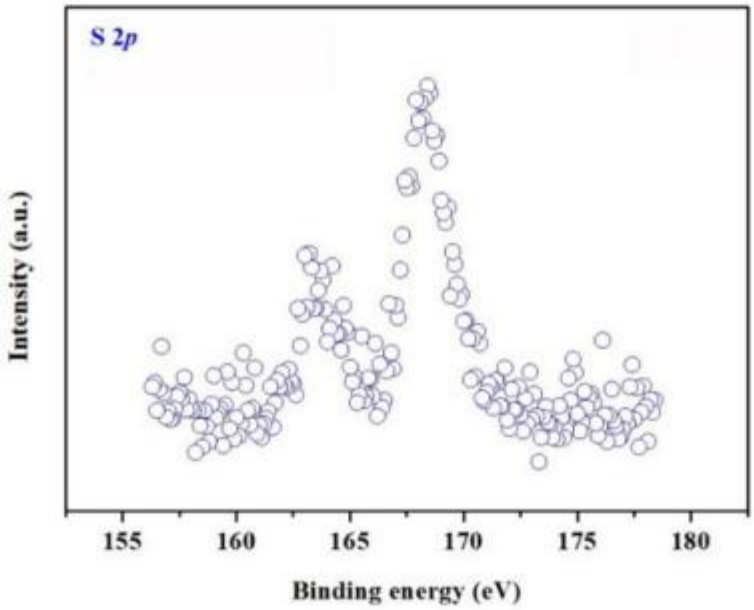

Figure S16. High-resolution S 2*p* XPS signal of COF-Fe/MXene-F_{17}.

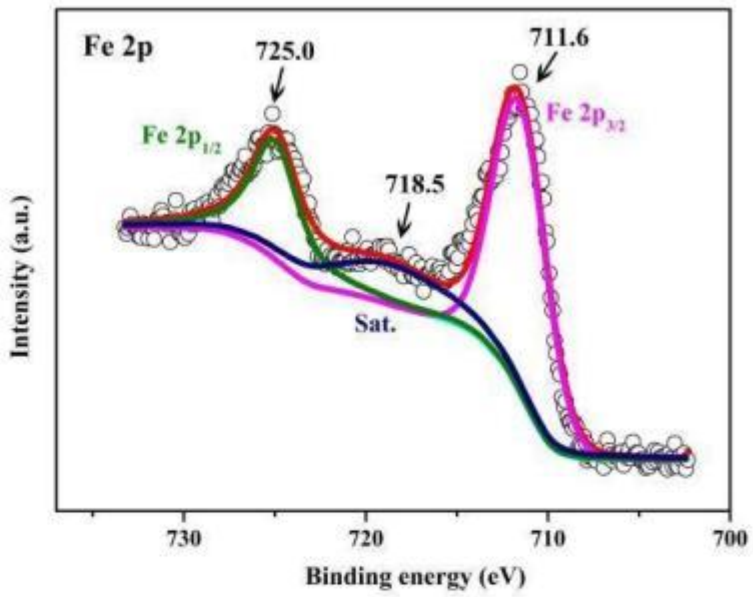

Figure S17. High-resolution Fe 2*p* XPS spectrum of COF-Fe/MXene-F_{17}.

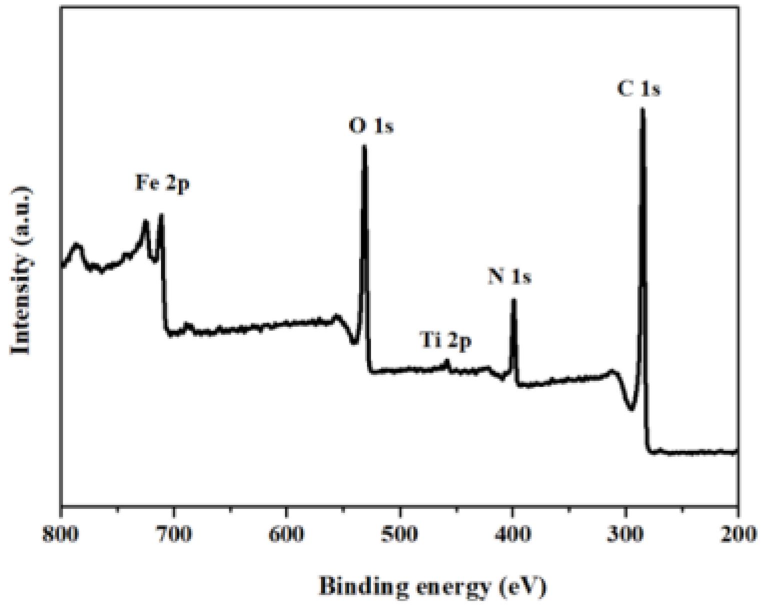

Figure S18. Full XPS spectrum of COF-Fe/MXene-F_{17} without 4-aminostyrene.

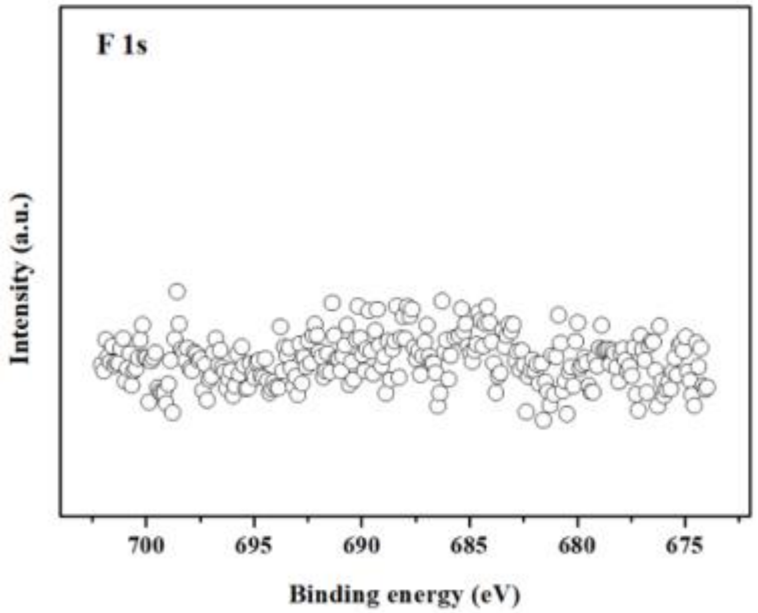

Figure S19. High-resolution F 1*s* XPS signal of COF-Fe/MXene-F_{17} without 4-aminostyrene.

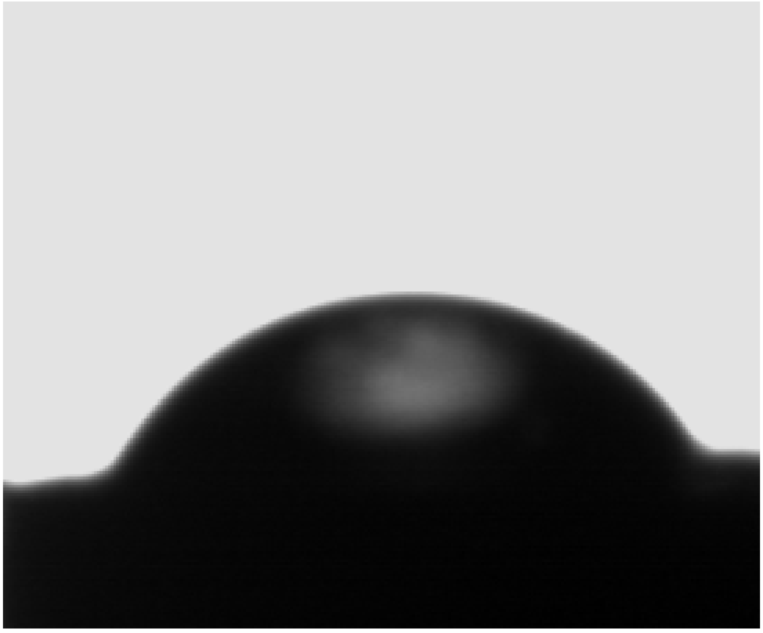

Figure S20. Water contact angle photo of COF-Fe/MXene-F_{17} without 4-aminostyrene.

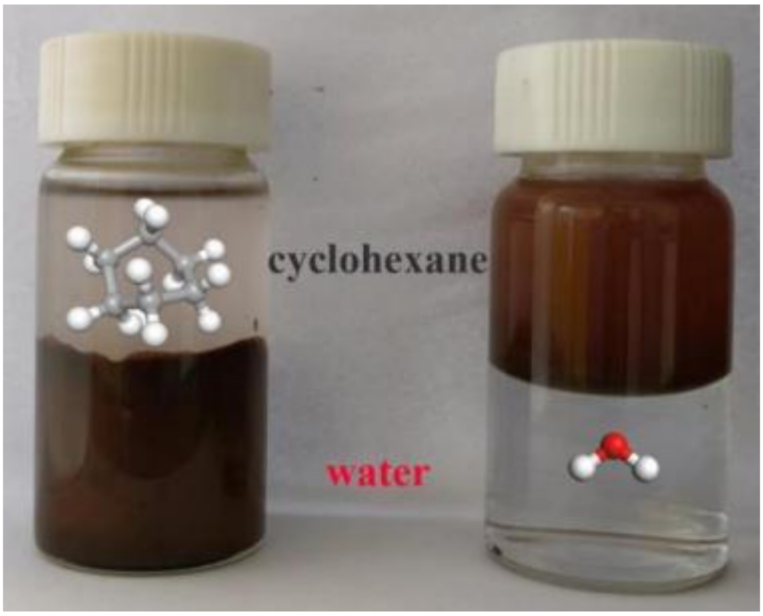

Figure S21. Photographs of COF-Fe/MXene (left) and COF-Fe/MXene-F_{17} (right) dispersed in the water-cyclohexane (v:v = 1:1) biphasic system.

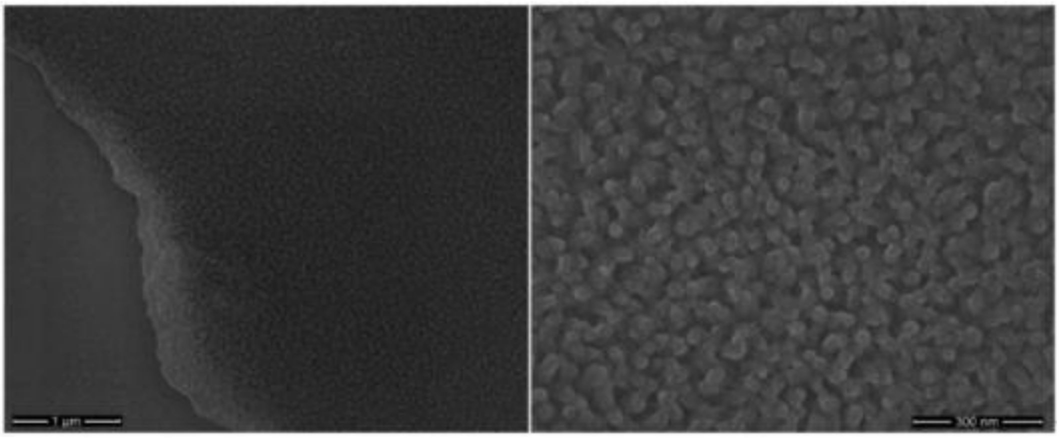

Figure S22. SEM images of COF/MXene.

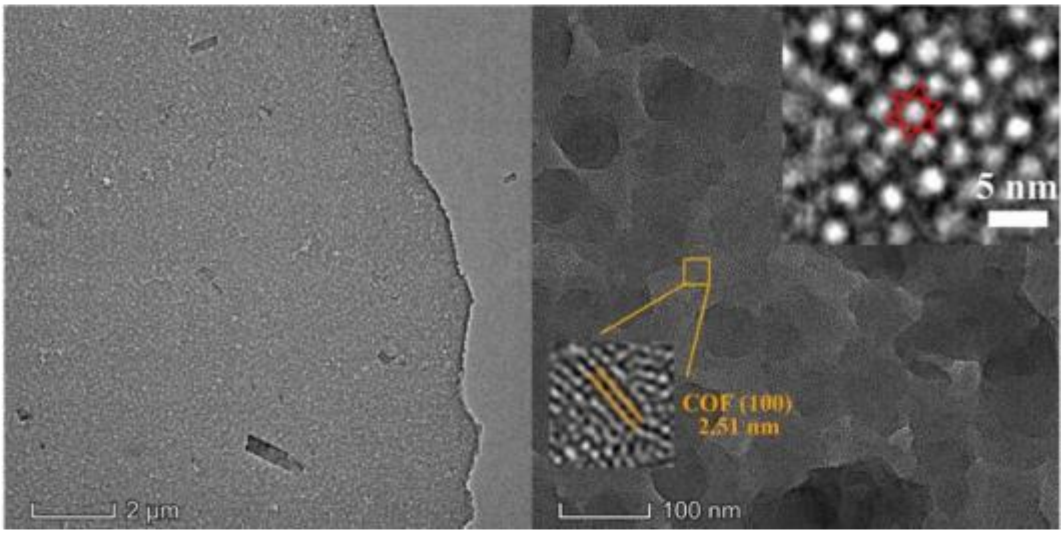

Figure S23. TEM images of the as-synthesized COF nanosheet.

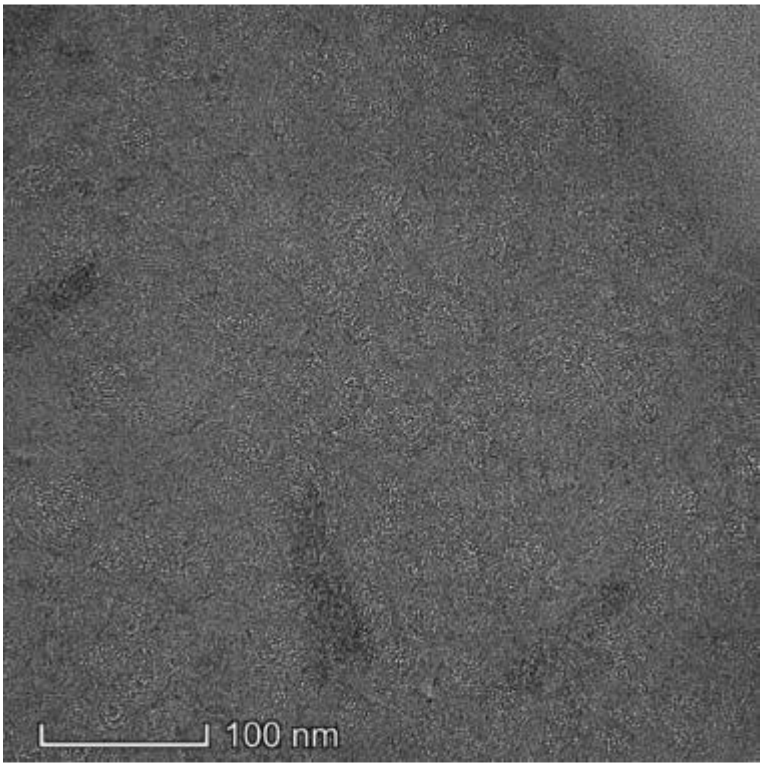

Figure S24. TEM image of the as-synthesized COF-Fe/MXene.

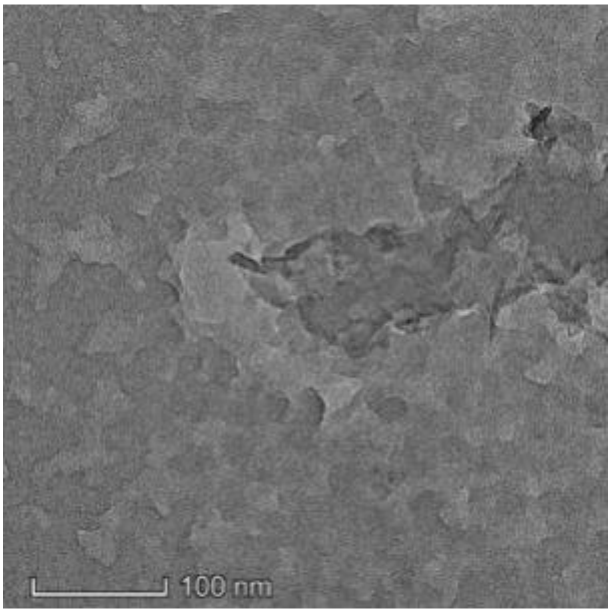

Figure S25. TEM image of the as-synthesized COF-Fe/MXene-F_{17}.

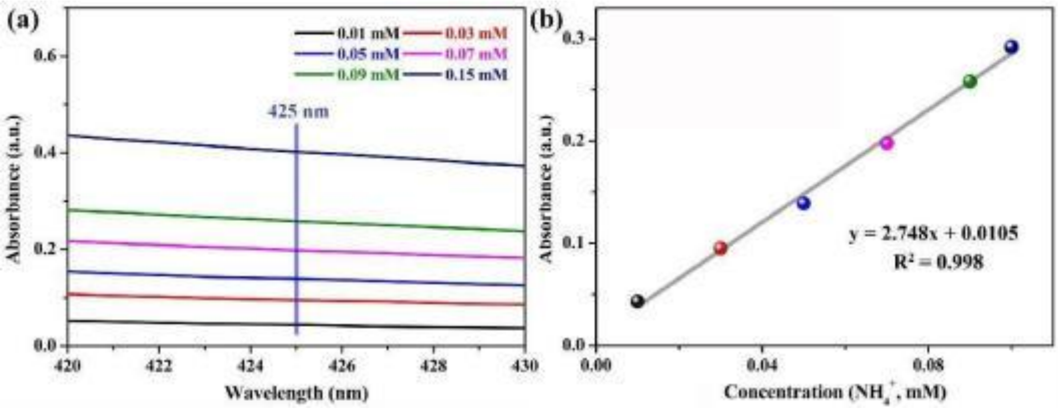

Figure S26. a) UV-vis absorption spectra of standard Nessler's reagent solutions. b) The calibration curve used for the estimation of NH_4^+ concentration.

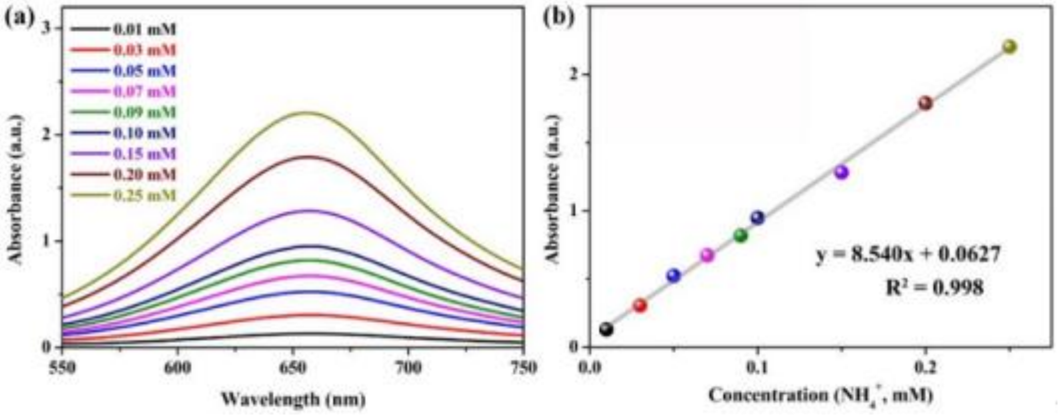

Figure S27. a) UV-vis absorption spectra of indophenol blue solutions. b) The calibration curve used for the estimation of NH_4^+ concentration.

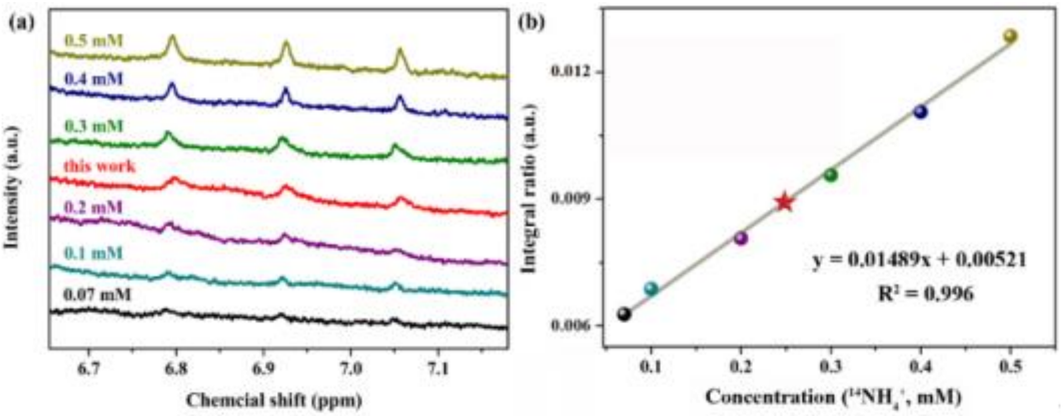

Figure S28. a) ^{1}H NMR spectra of ammonium solutions. b) The calibration curve used for the estimation of $^{14}NH_4^+$ concentration.

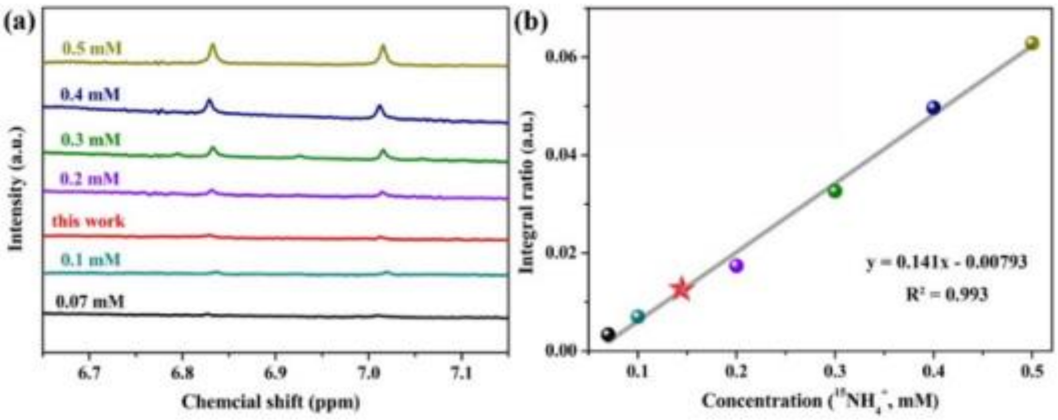

Figure S29. a) ^{1}H NMR spectra of ammonium solutions. b) The calibration curve used for the estimation of $^{15}NH_4^+$ concentration.

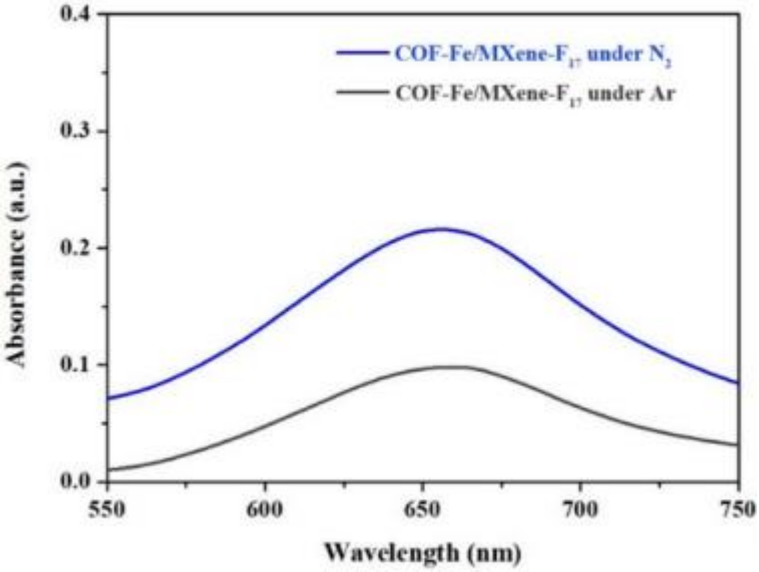

Figure S30. UV-vis absorption spectra of N_2-saturated (blue) and Ar-saturated (gray) electrolytes at –0.5 V versus RHE using the indophenol blue method.

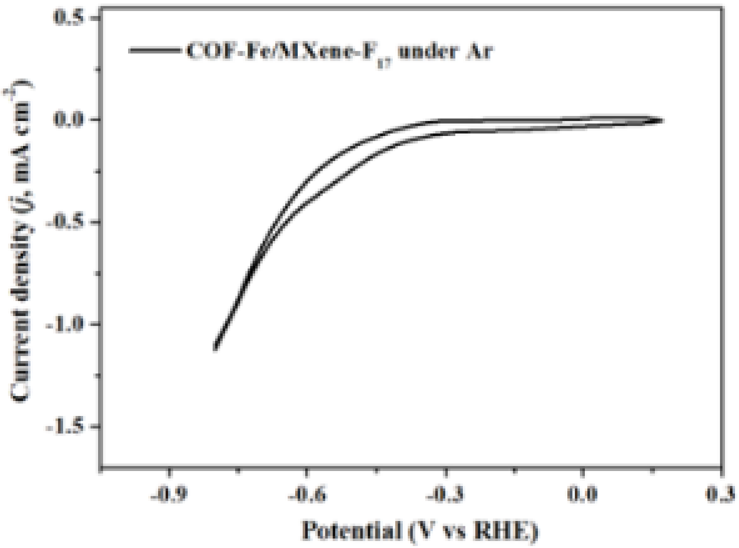

Figure S31. CV curves of COF-Fe/MXene-F_{17} under the Ar atmosphere.

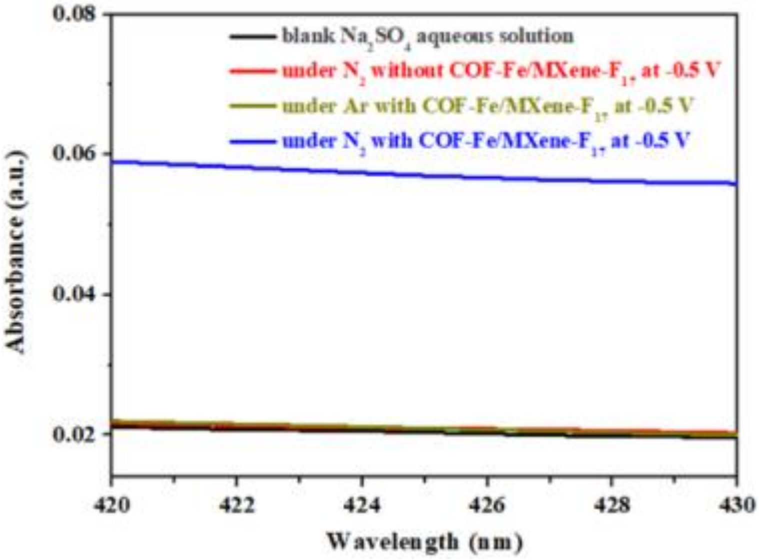

Figure S32. UV-vis absorption spectra of the 0.1 M Na_2SO_4 water solution, N_2-saturated post electrolyte at –0.5 V versus RHE without COF-Fe/MXene-F_{17} modified electrode, and N_2- or Ar-saturated post electrolytes at –0.5 V versus RHE with COF-Fe/MXene-F_{17} modified electrode.

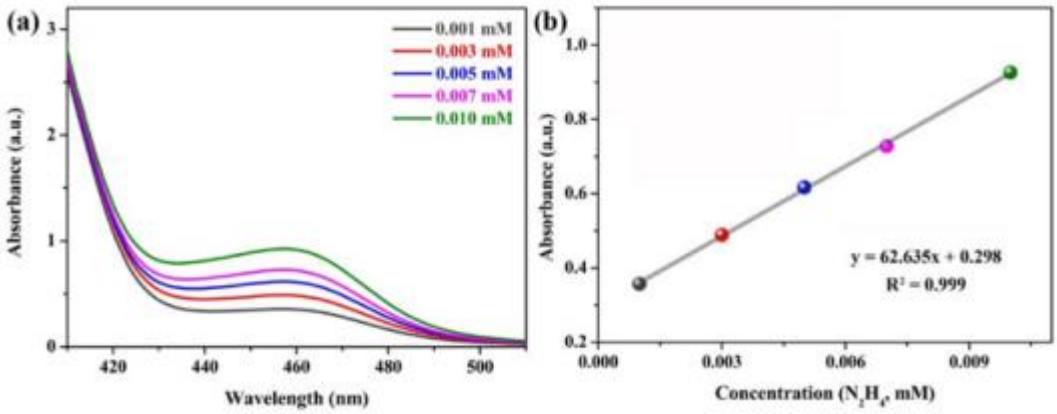

Figure S33. a) UV-vis absorption spectra. b) The calibration curve for calculating hydrazine concentration using the Watt and Chrisp approach.

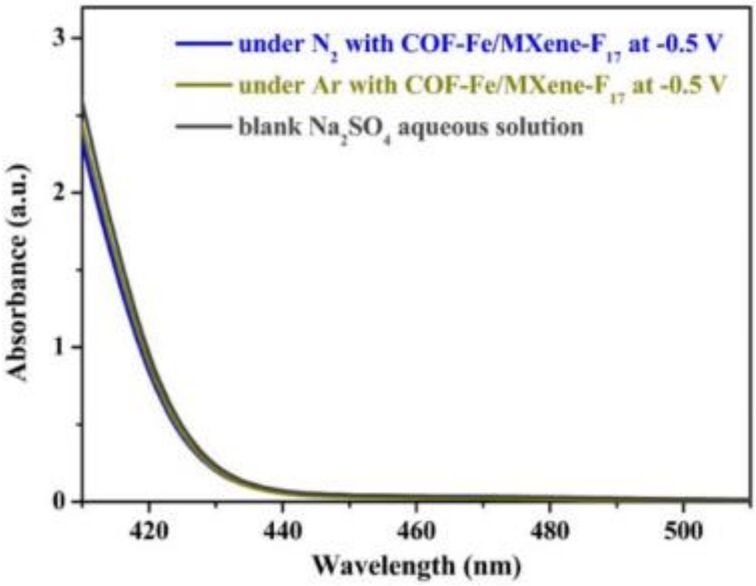

Figure S34. UV-vis absorption spectra of the blank Na_2SO_4 electrolyte and post electrolytes after NRR measurements at –0.5 V versus RHE with COF-Fe/MXene-F_{17} modified electrode under Ar- and N_2-saturated conditions.

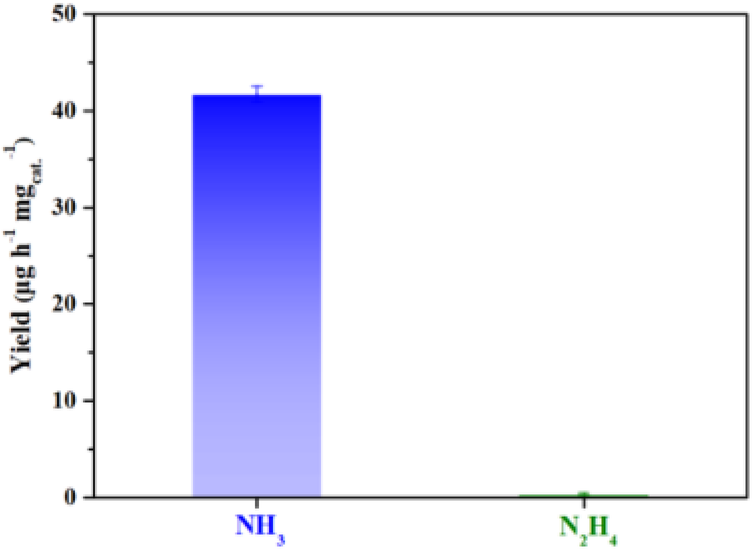

Figure S35. NH_3 and N_2H_4 yields during the electrochemical NRR process with COF-Fe/MXene-F_{17} modified electrode at –0.5 V versus RHE under N_2-saturated atmosphere.

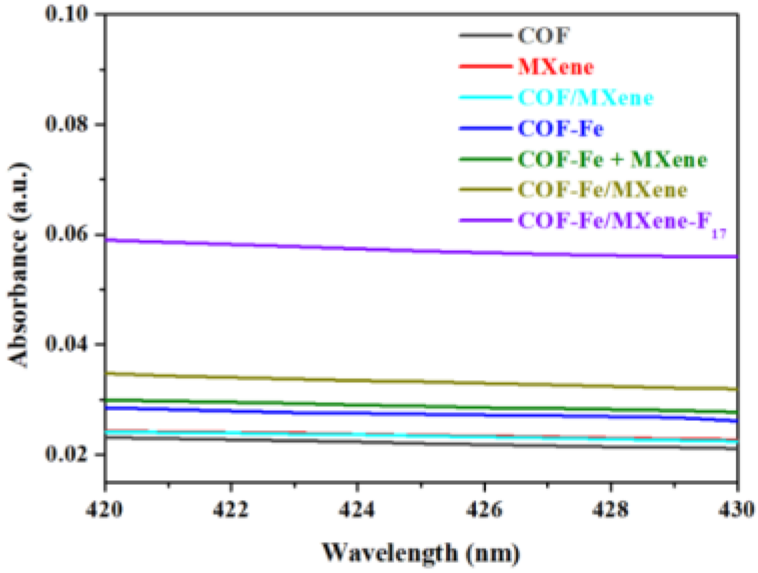

Figure S36. UV-vis absorption spectra of N_2-saturated post electrolytes at –0.5 V versus RHE with electrodes modified by different materials.

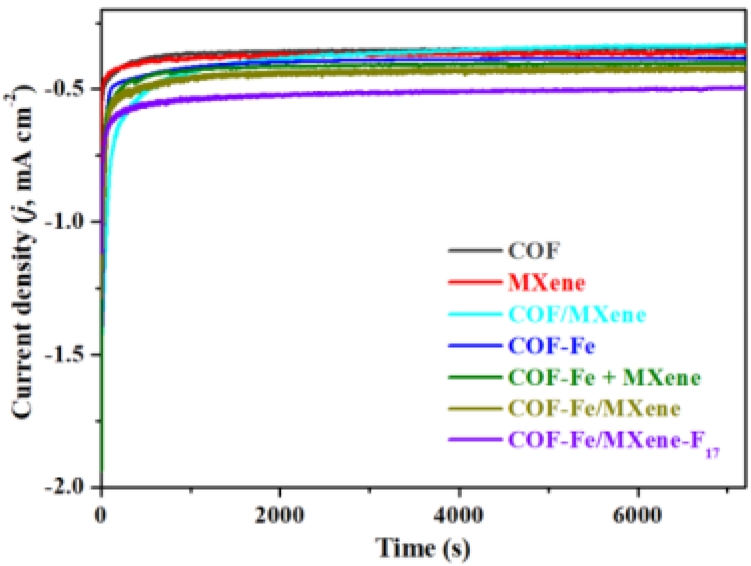

Figure S37. CA curves in N_2-saturated atmosphere at –0.5 V versus RHE.

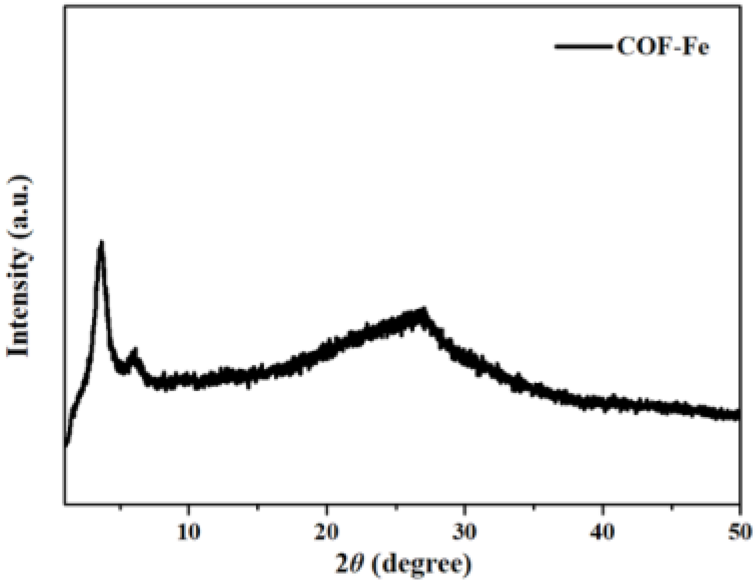

Figure S38. PXRD pattern of COF-Fe.

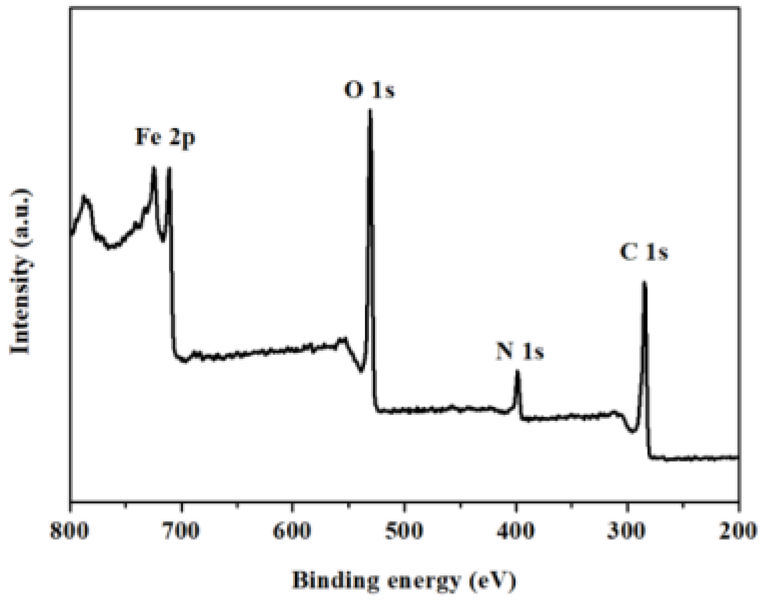

Figure S39. Full XPS spectrum of COF-Fe.

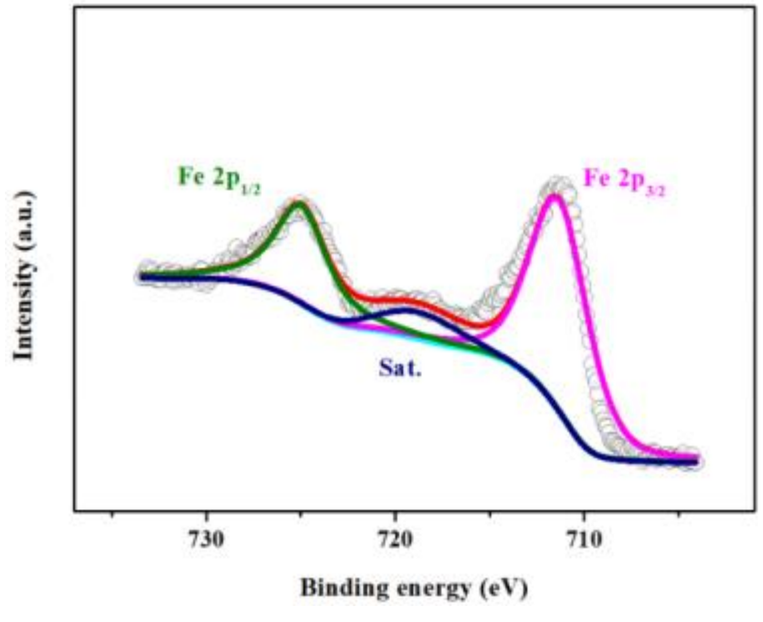

Figure S40. High-resolution Fe 2*p* XPS spectrum of COF-Fe.

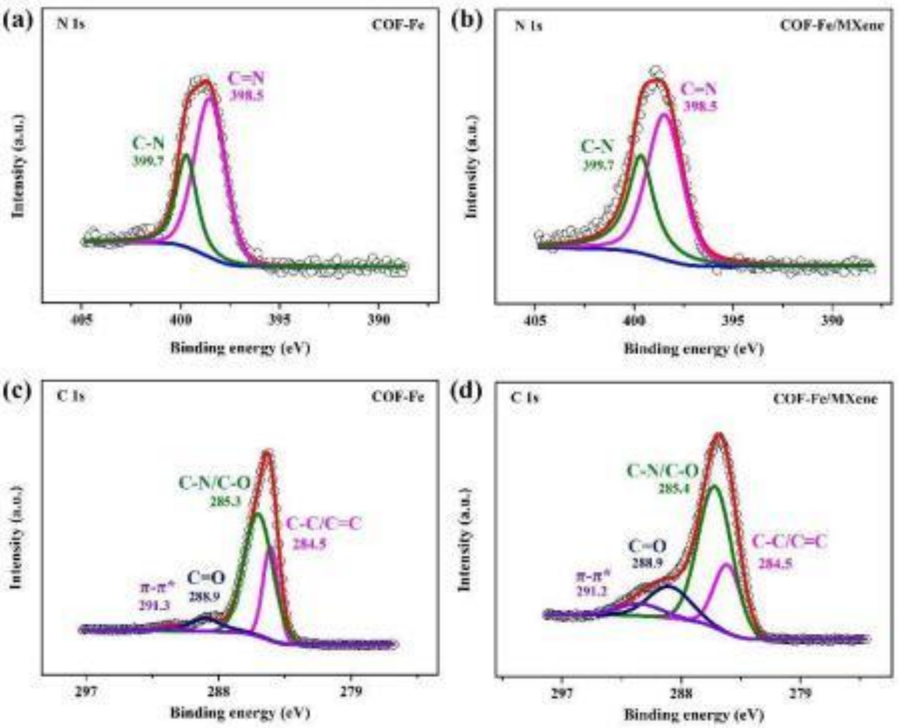

Figure S41. High-resolution C 1*s* and N 1*s* XPS spectra of COF-Fe and COF-Fe/MXene.

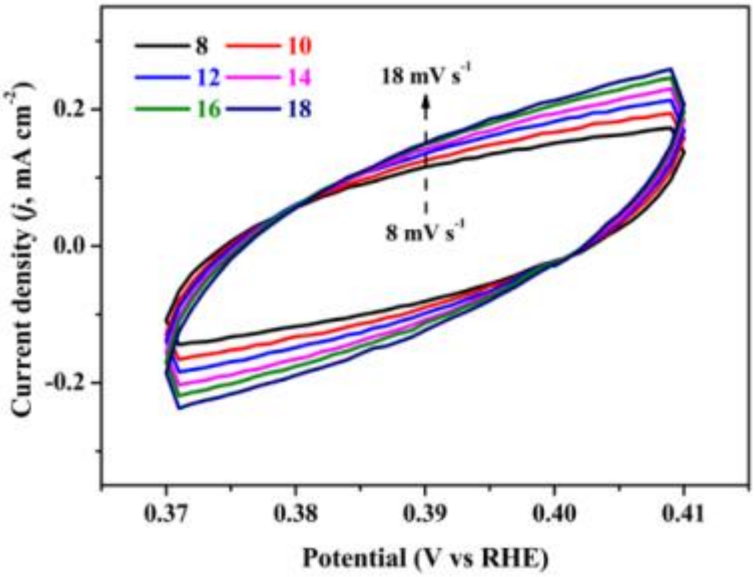

Figure S42. CV curves of the alkylamino group modified MXene at various scan rates.

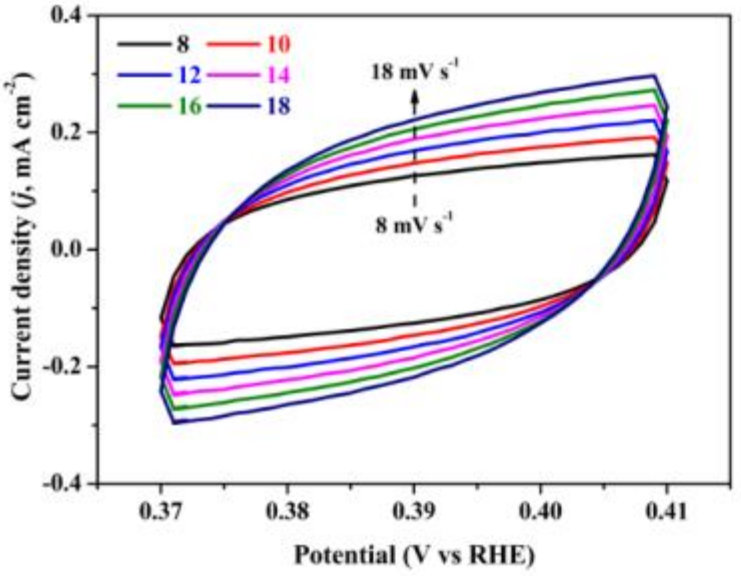

Figure S43. CV curves of COF-Fe at various scan rates.

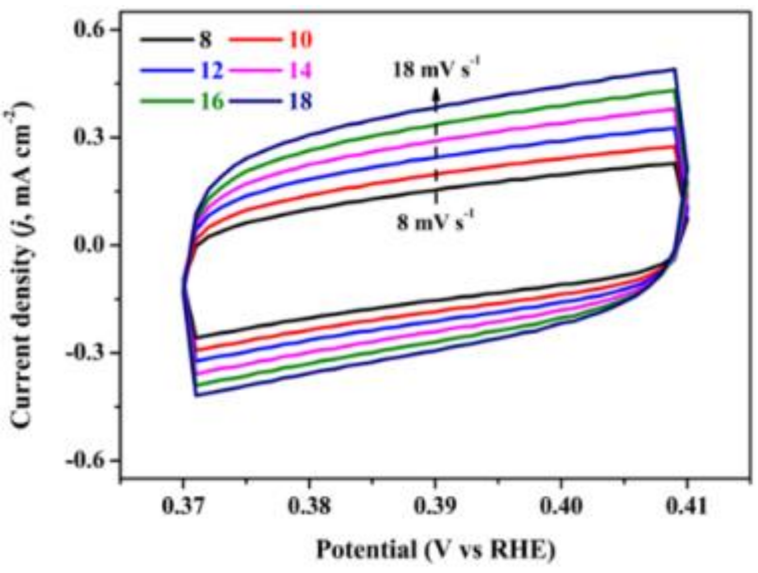

Figure S44. CV curves of COF-Fe/MXene at various scan rates.

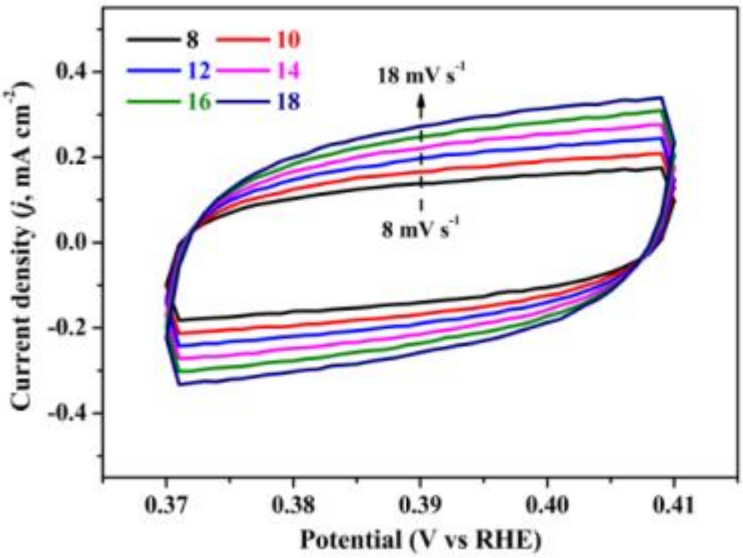

Figure S45. CV curves of COF-Fe + MXene at various scan rates.

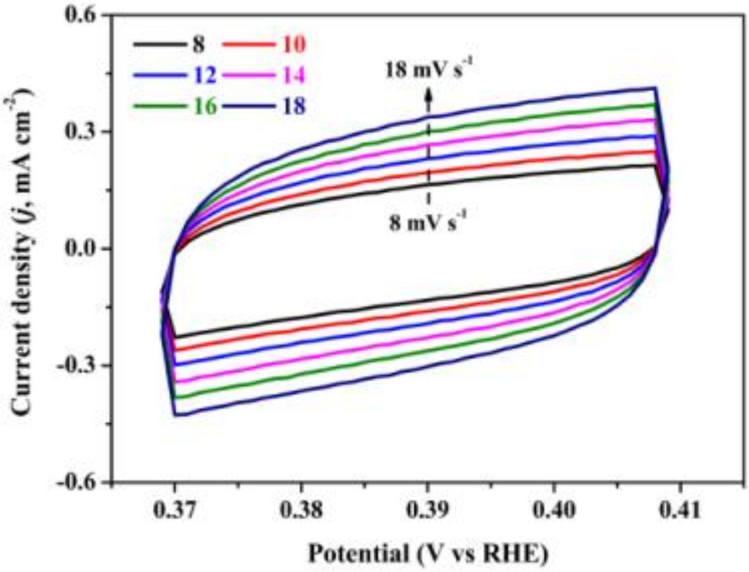

Figure S46. CV curves of COF-Fe/MXene-F_{17} at various scan rates.

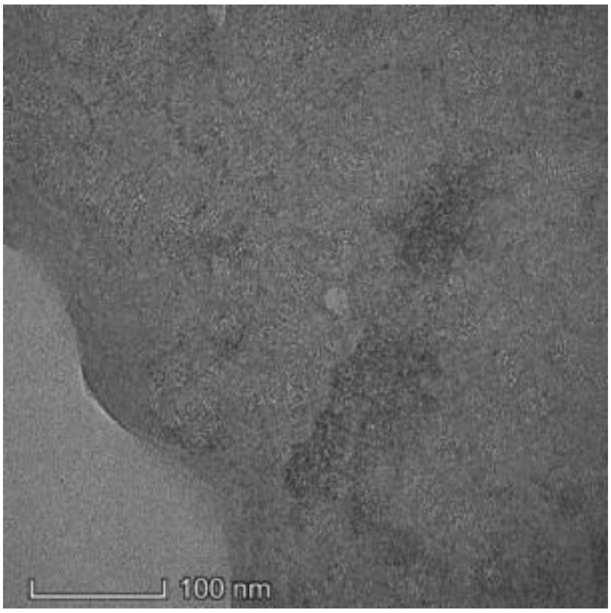

Figure S47. TEM image of COF-Fe/MXene-C_3.

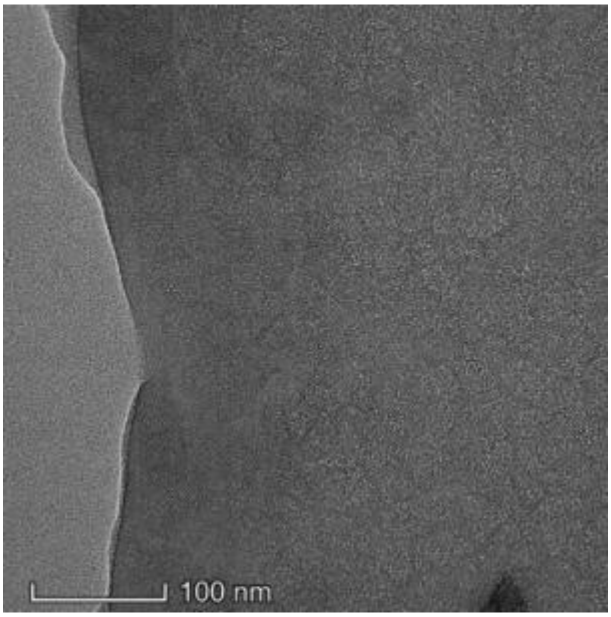

Figure S48. TEM image of COF-Fe/MXene-C_6.

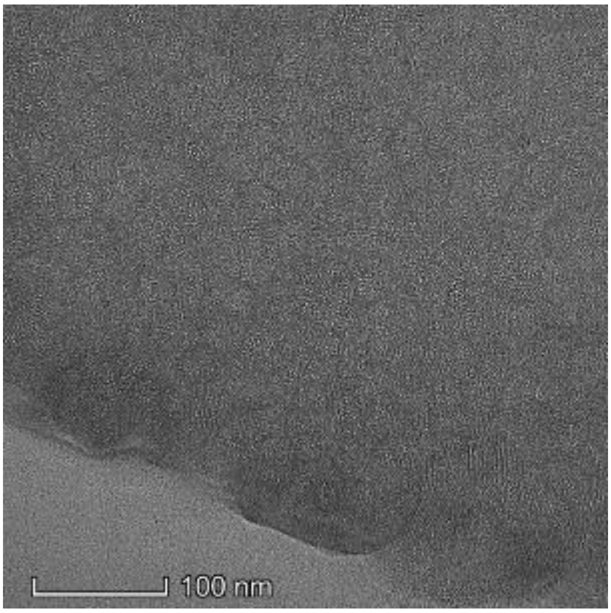

Figure S49. TEM image of COF-Fe/MXene-C_{18}.

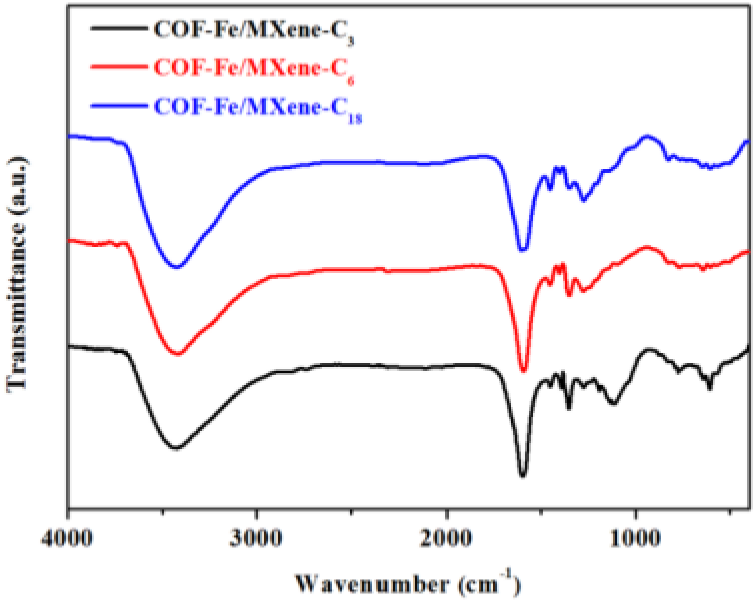

Figure S50. FT-IR spectra of COF-Fe/MXene-C_3, COF-Fe/MXene-C_6, and COF-Fe/MXene-C_{18}.

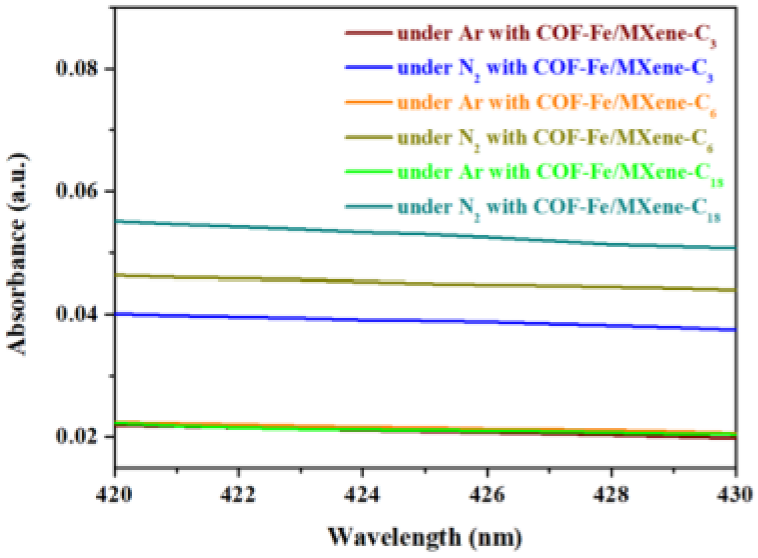

Figure S51. UV-vis absorption spectra of N_2- and Ar-saturated post electrolytes at –0.5 V versus RHE for 2 h with COF-Fe/MXene-C_3, COF-Fe/MXene-C_6, and COF-Fe/MXene-C_{18} modified electrodes.

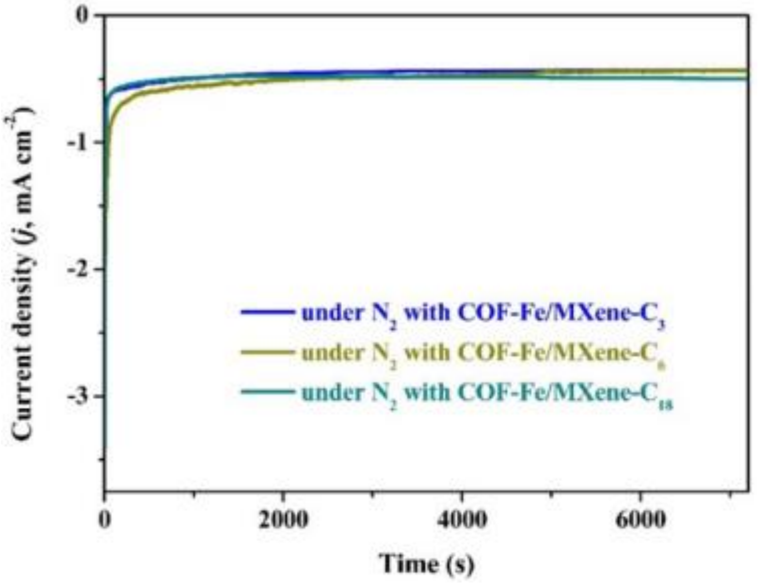

Figure S52. CA curves of COF-Fe/MXene-C_3, COF-Fe/MXene-C_6, and COF-Fe/MXene-C_{18} in N_2-saturated atmosphere at –0.5 V versus RHE.

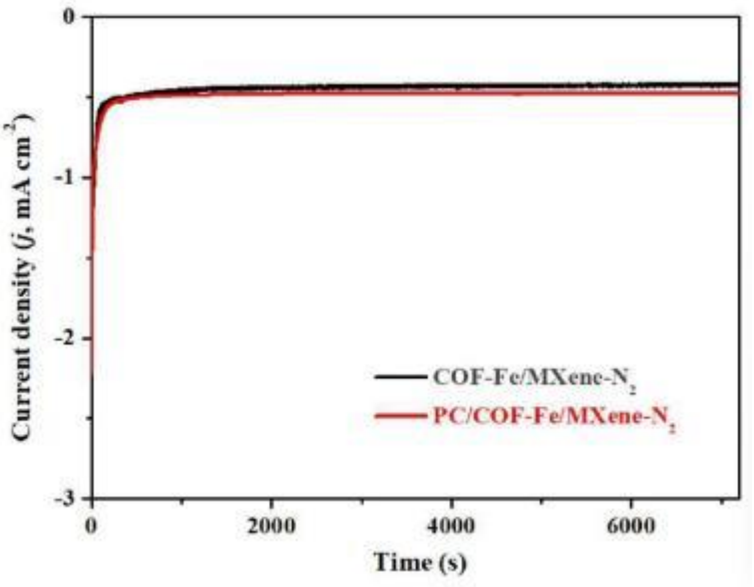

Figure S53. CA curves of PC/COF-Fe/MXene and COF-Fe/MXene in the N_2-saturated atmosphere at –0.5 V versus RHE.

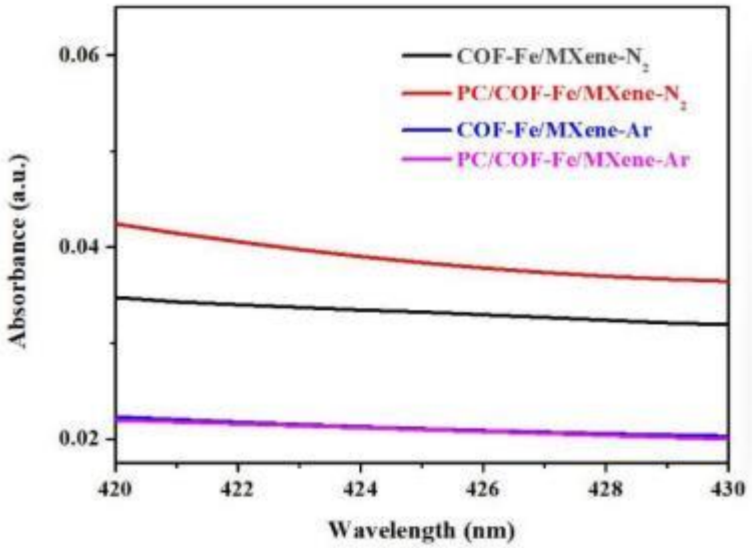

Figure S54. UV-vis absorption spectra of the N_2-saturated post electrolytes at –0.5 V versus RHE for 2 h with PC/COF-Fe/MXene and COF-Fe/MXene modified electrodes.

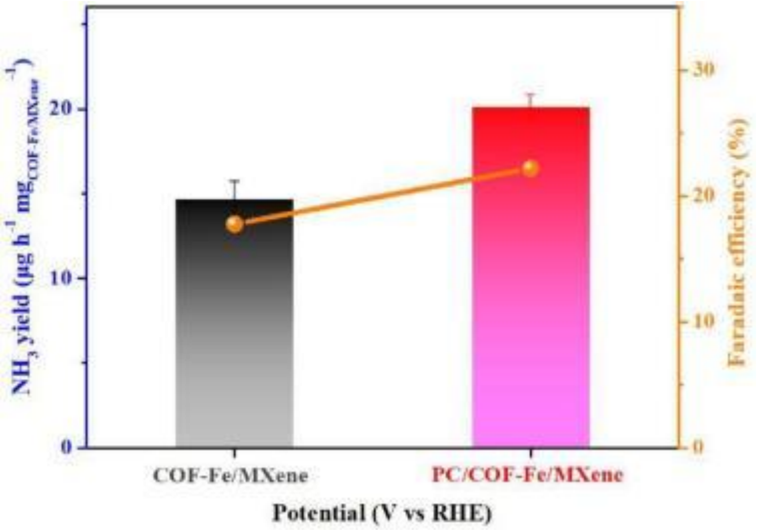

Figure S55. NRR performances of PC/COF-Fe/MXene and COF-Fe/MXene in the N_2-saturated atmosphere at –0.5 V versus RHE.

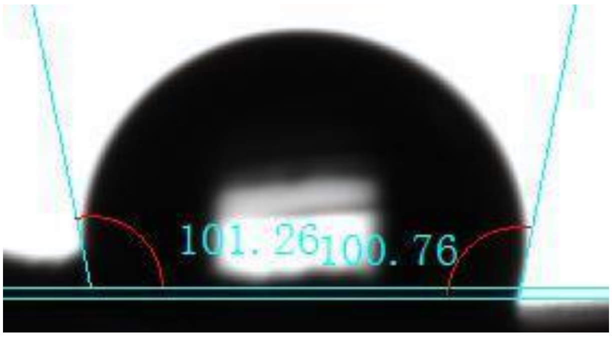

Figure S56. Water contact angle photo of PC/COF-Fe/MXene.

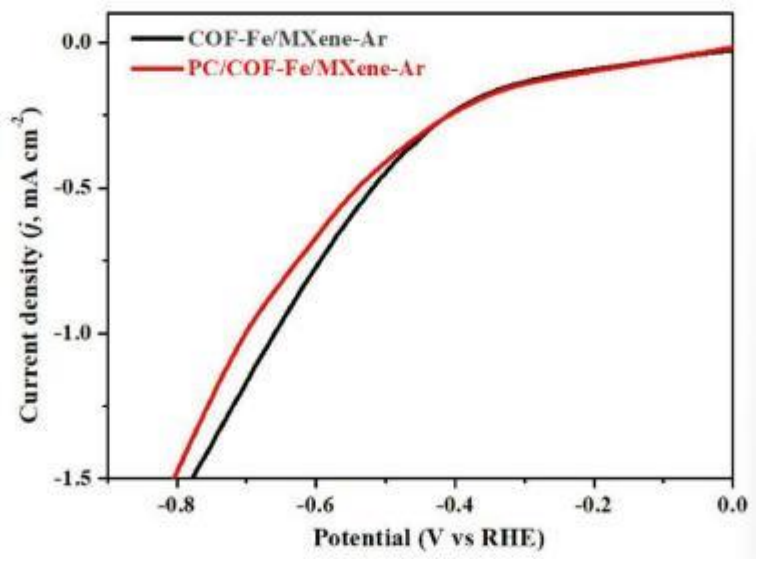

Figure S57. LSV curves under the Ar atmosphere of PC/COF-Fe/MXene and COF-Fe/MXene.

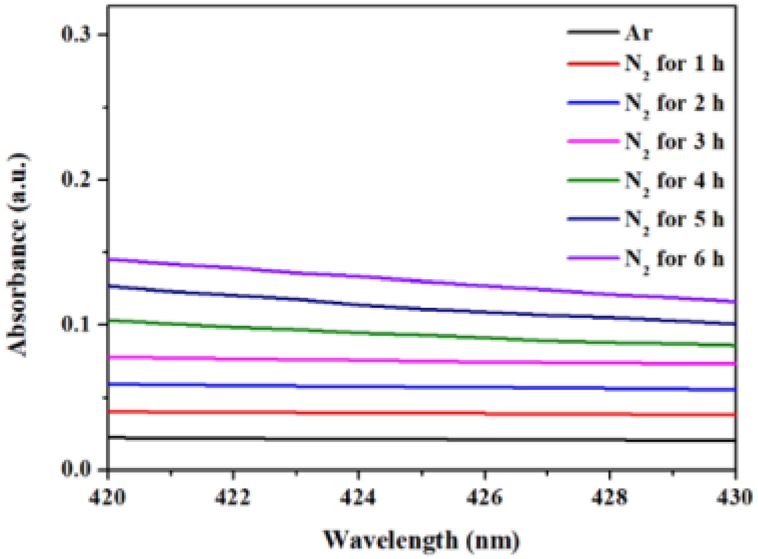

Figure S58. UV-vis absorption spectra of N_2-saturated post electrolytes at –0.5 V versus RHE for different durations with COF-Fe/MXene-F_{17} modified electrode.

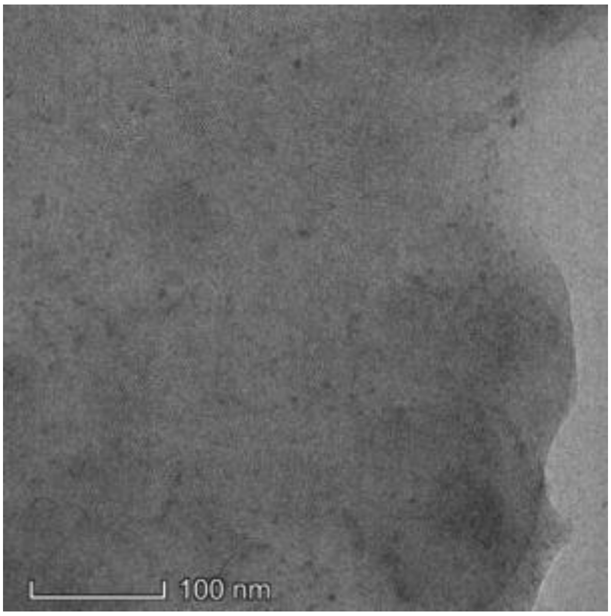

Figure S59. TEM image of used COF-Fe/MXene-F_{17}.

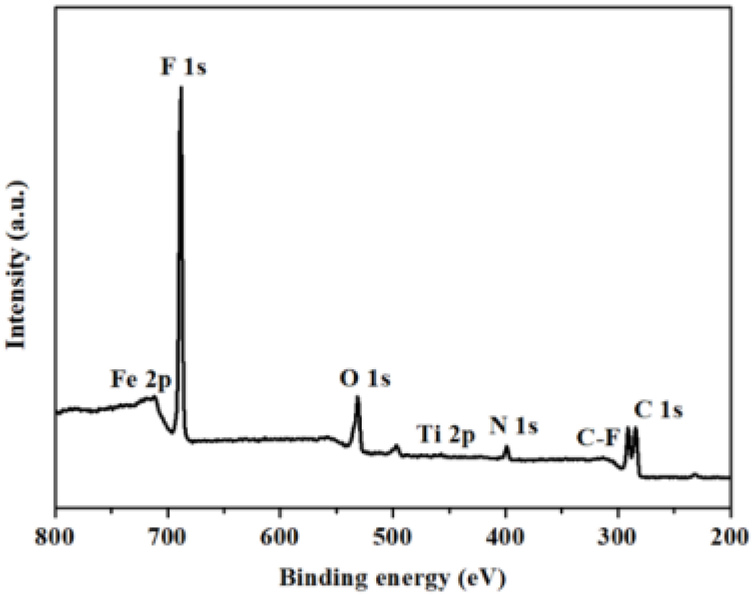

Figure S60. Full XPS spectrum of used COF-Fe/MXene-F_{17}.

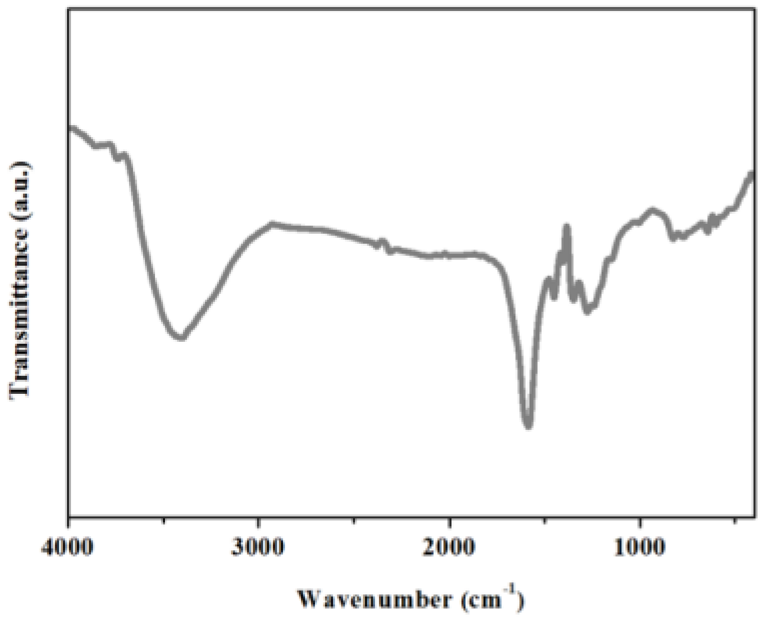

Figure S61. FT-IR spectrum of used COF-Fe/MXene-F_{17}.

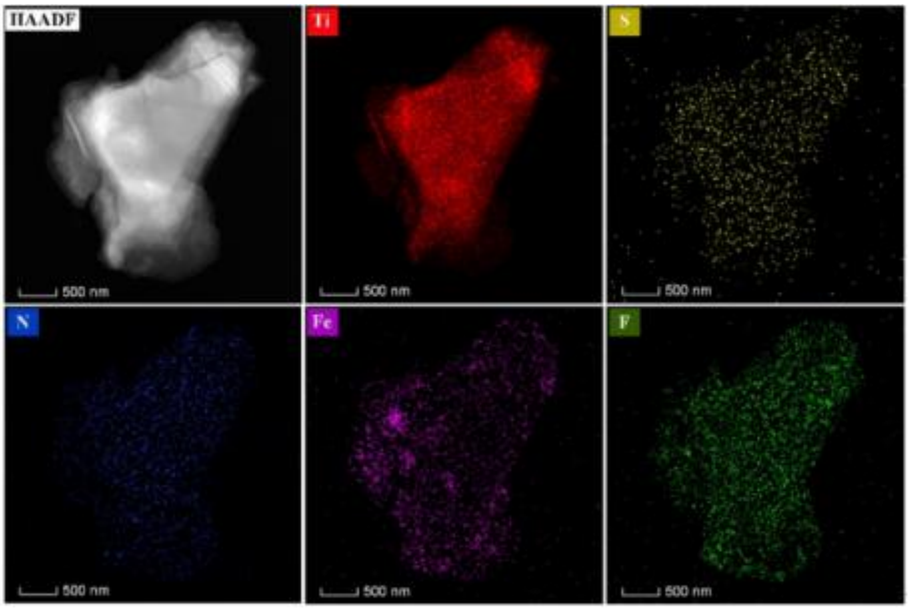

Figure S62. HAADF-STEM and EDS mappings of used COF-Fe/MXene-F_{17}.

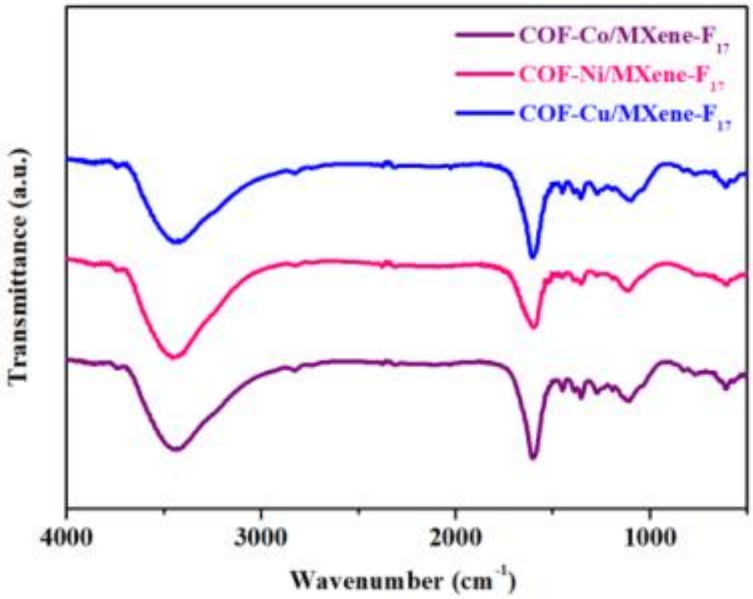

Figure S63. FT-IR spectra of COF-Co/MXene-F_{17}, COF-Ni/MXene-F_{17}, and COF-Cu/MXene-F_{17}.

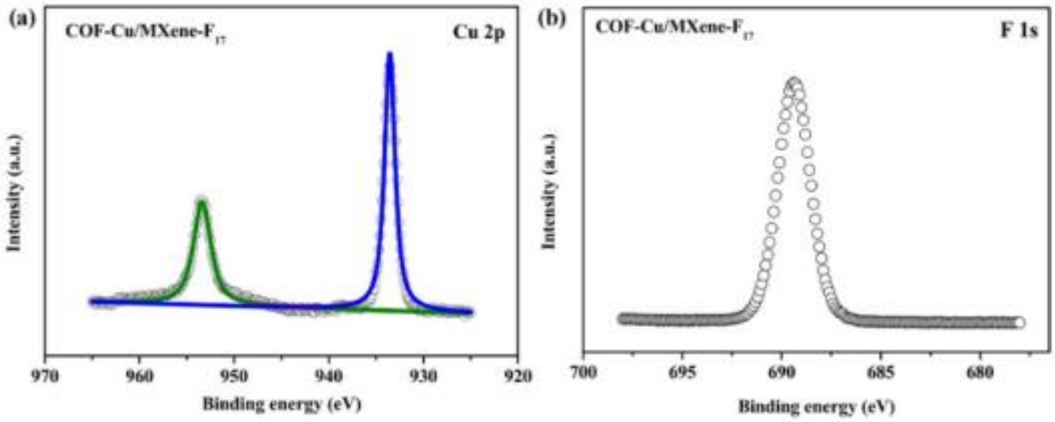

Figure S64. High-resolution Cu 2*p* and F 1*s* XPS spectra of COF-Cu/MXene-F_{17}.

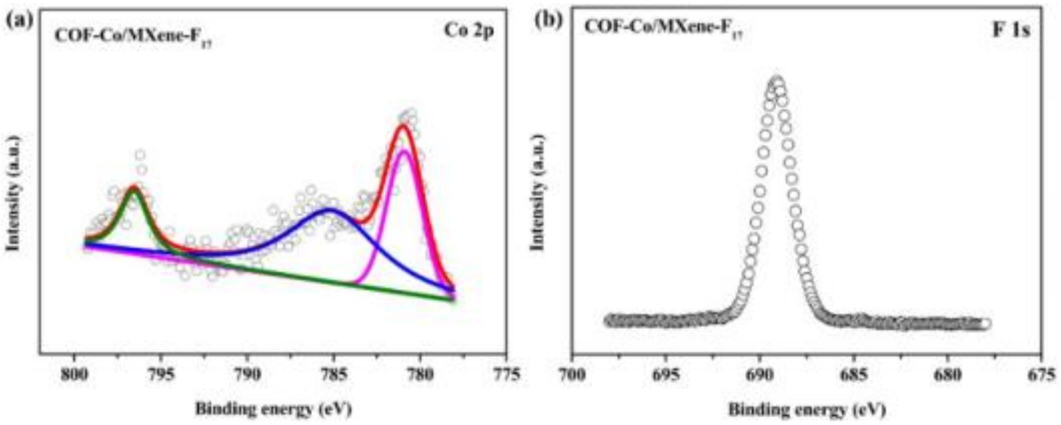

Figure S65. High-resolution Co 2*p* and F 1*s* XPS spectra of COF-Co/MXene-F_{17}.

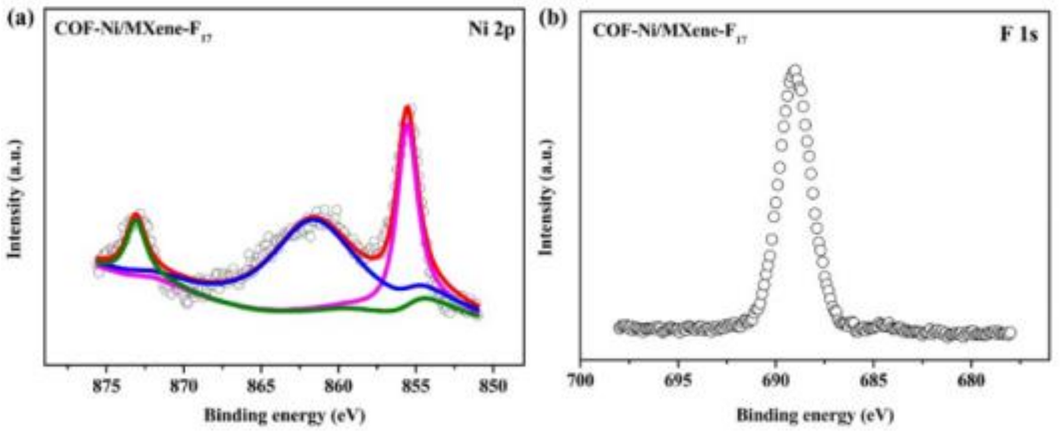

Figure S66. High-resolution Ni 2*p* and F 1*s* XPS spectra of COF-Ni/MXene-F_{17}.

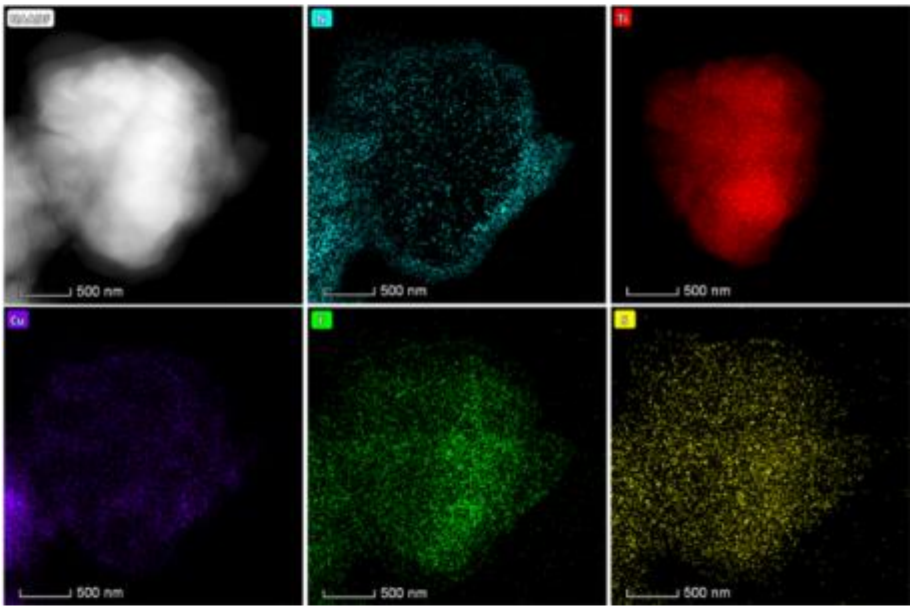

Figure S67. HAADF-STEM and EDS mappings of COF-Cu/MXene-F_{17}.

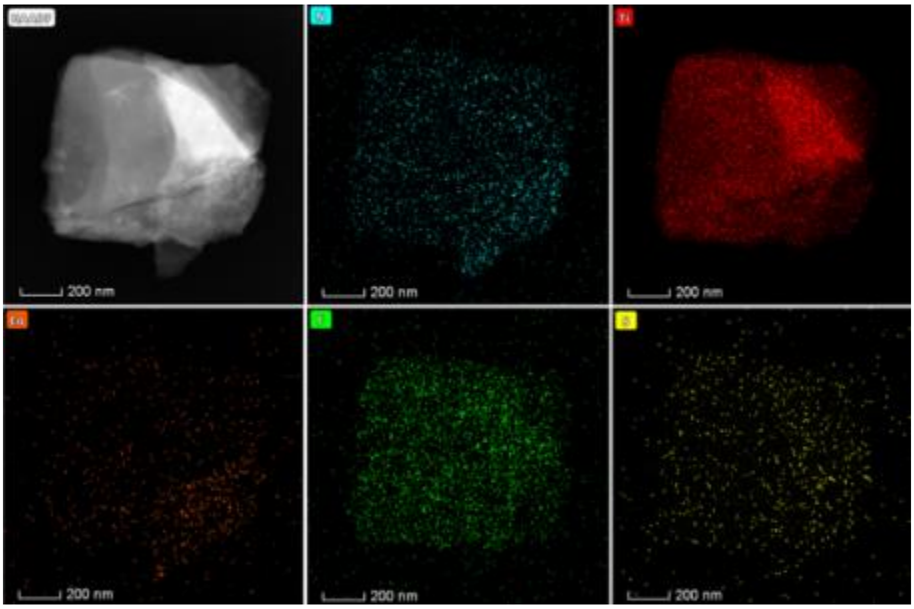

Figure S68. HAADF-STEM and EDS mappings of COF-Co/MXene-F_{17}.

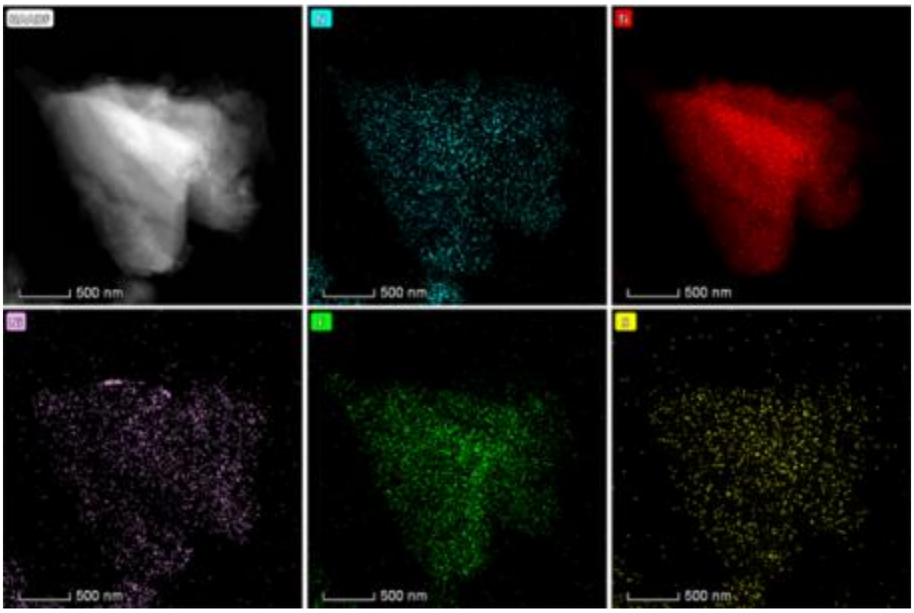

Figure S69. HAADF-STEM and EDS mappings of COF-Ni/MXene-F_{17}.

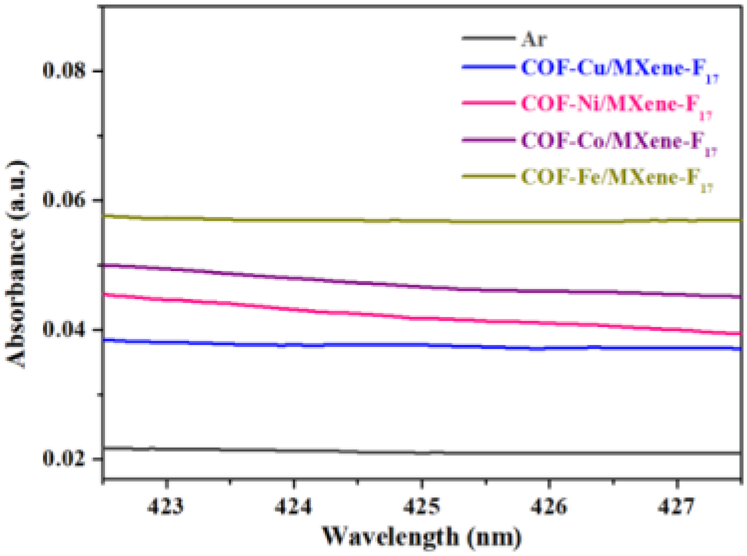

Figure S70. UV-vis absorption spectra of N_2-saturated post electrolytes at –0.5 V versus RHE for 2 h with COF-Cu/MXene-F_{17}, COF-Co/MXene-F_{17}, COF-Ni/MXene-F_{17}, and COF-Fe/MXene-F_{17} modified electrodes.

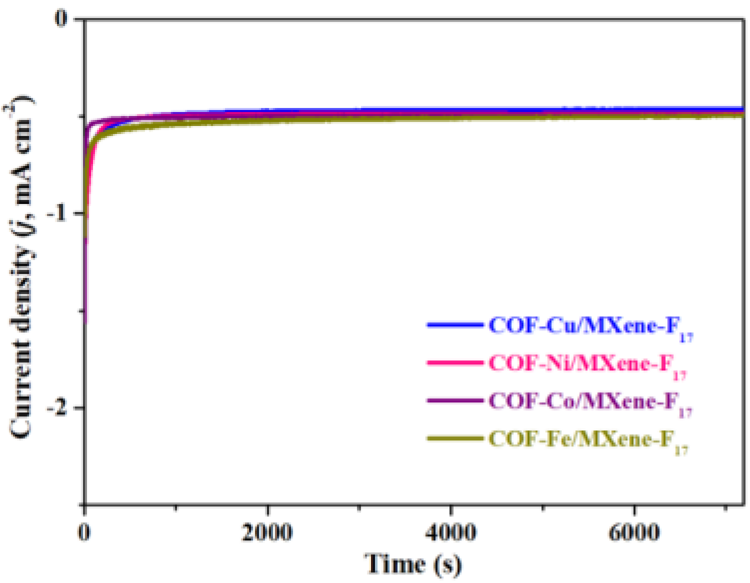

Figure S71. CA curves of COF-Cu/MXene-F_{17}, COF-Co/MXene-F_{17}, COF-Ni/MXene-F_{17}, and COF-Fe/MXene-F_{17} in N_2-saturated atmosphere at –0.5 V versus RHE.

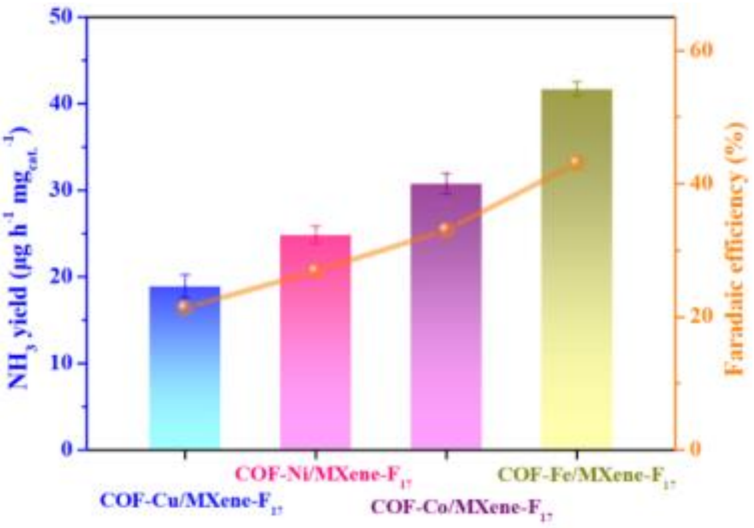

Figure S72. NRR performances of COF-Cu/MXene-F_{17}, COF-Co/MXene-F_{17}, COF-Ni/MXene-F_{17}, and COF-Fe/MXene-F_{17} in N_2-saturated atmosphere at –0.5 V versus RHE.

Section S3. Supplementary Table

Table S1. Summary of the NRR performances for some representative Fe-based catalysts

under mild conditions.

Catalyst	Electrolyte	Potential (V versus RHE)	Faradaic efficiency (%)	NH_3 yield (μg h^{-1} $mg_{cat.}^{-1}$)	Ref.
Fe_2O_3 nanorod	0.1 M Na_2SO_4	–0.8	0.94	15.9	[6]
Fe-N/C-CNT	0.1 M KOH	–0.2	9.28	34.83	[7]
Fe_3O_4@rGO	0.1 M Na_2SO_4	–0.3	11.47	28.01	[8]
$FeVO_4$	0.5 M $LiClO_4$	–0.4	13.8	48.5	[9]
Fe-$InVO_4$	0.1 M HCl	–0.4	14.27	17.23	[10]
FeOOH QDs-GS	0.1 M $LiClO_4$	–0.4	14.6	27.3	[11]
Fe-CeO_2	0.5 M $LiClO_4$	–0.5	14.7	26.2	[12]
FeTPPCl	0.1 M Na_2SO_4-PBS	–0.3	16.76	18.28	[13]
OPA-PCN-222(Fe)	0.1 M HCl	–0.4	17.2	22.1	[14]
ISAS-Fe/NC	0.1 M PBS	–0.4	18.6	62.9	[15]
FeMoPPc	0.1 M KOH	–0.3	20.62	36.33	[16]
FePCTa-PPy	0.1 M Na_2SO_4	–0.3	22.5	47.49	[17]
Fe-TiO_2	0.5 M $LiClO_4$	–0.4	25.6	25.47	[18]
MoFe@NG	0.25 M $LiClO_4$	–0.2	41.7	3.45	[19]
**COF-Fe/MXene-F_{17}**	**0.1 M Na_2SO_4**	**–0.5**	**43.1**	**41.8**	**this work**

Section S4. References

[1] K. Dey, M. Pal, K. C. Rout, S. Kunjattu H, A. Das, R. Mukherjee, U. K. Kharul, R. Banerjee, *J. Am. Chem. Soc.* **2017**, *139*, 13083.

[2] Y. C. Hao, Y. Guo, L. W. Chen, M. Shu, X. Y. Wang, T. A. Bu, W. Y. Gao, N. Zhang, X. Su, X. Feng, J. W. Zhou, B. Wang, C. W. Hu, A. X. Yin, R. Si, Y. W. Zhang, C. H. Yan, *Nat. Catal.* **2019**, *2*, 448.

[3] J. Wang, L. Yu, L. Hu, G. Chen, H. Xin, X. Feng, *Nat. Commun.* **2018**, *9*, 1795.

[4] A. C. Nielander, J. M. McEnaney, J. A. Schwalbe, J. G. Baker, S. J. Blair, L. Wang, J. G. Pelton, S. Z. Andersen, K. Enemark-Rasmussen, V. Čolić, S. Yang, S. F. Bent, M. Cargnello, J. Kibsgaard, P. C. K. Vesborg, I. Chorkendorff, T. F. Jaramillo, *ACS Catal.* **2019**, *9*, 5797.

[5] W. Xu, G. Fan, J. Chen, J. Li, L. Zhang, S. Zhu, X. Su, F. Cheng, J. Chen, *Angew. Chem. Int. Ed.* **2020**, *59*, 3511.

[6] X. Xiang, Z. Wang, X. Shi, M. Fan, X. Sun, *ChemCatChem* **2018**, *10,* 4530.

[7] Y. Wang, X. Cui, J. Zhao, G. Jia, L. Gu, Q. Zhang, L. Meng, Z. Shi, L. Zheng, C. Wang, Z. Zhang, W. Zheng, *ACS Catal.* **2019**, *9*, 336.

[8] F. Wang, L. Xia, X. Li, W. Yang, Y. Zhao, J. Mao, *Energy Environ. Mater*. **2021**, *4*, 88.

[9] P. Shen, Y. Liu, Q. Li, K. Chu, *Chem. Commun*. **2020**, *56*, 10505.

[10] J. Li, M. Zheng, F. Wei, C. Dong, Z. Xiu, W. Mu, X. Zhou, Y. Ding, X. Han, *Chem. Eng. J.* **2022**, *431*, 133383.

[11] X. Zhu, J. Zhao, L. Ji, T. Wu, T. Wang, S. Gao, A. A. Alshehri, K. A. Alzahrani, Y. Luo, Y. Xiang, B. Zheng, X. Sun, *Nano Res.* **2020**, *13,* 209.

[12] K. Chu, Y.-H. Cheng, Q.-Q. Li, Y.-P. Liu, Y. Tian, *J. Mater. Chem. A* **2020**, *8,* 5865.

[13] X. Yang, S. Sun, L. Meng, K. Li, S. Mukherjee, X. Chen, J. Lv, S. Liang, H.-Y. Zang, L.-K. Yan, G. Wu, *Appl. Catal. B-Environ.* **2021**, *285*, 119794.

[14] H. He, H.-K. Li, Q.-Q. Zhu, C.-P. Li, Z. Zhang, M. Du, *Appl. Catal. B-Environ.* **2022**, *316*, 121673.

[15] F. Lü, S. Zhao, R. Guo, J. He, X. Peng, H. Bao, J. Fu, L. Han, G. Qi, J. Luo, X. Tang, X. Liu, *Nano Energy* **2019**, *61,* 420.

[16] Y. Wang, W. Cheng, P. Yuan, G. Yang, S. Mu, J. Liang, H. Xia, K. Guo, M. Liu, S. Zhao, G. Qu, B.-A. Lu, Y. Hu, J. Hu, J.-N. Zhang, *Adv. Sci.* **2021**, 2102915.

[17] W. Qiu, N. Yang, D. Luo, J. Wang, L. Zheng, Y. Zhu, E. M. Akinoglu, Q. Huang, L. Shui, R. Wang, G. Zhou, X. Wang, Z. Chen, *Appl. Catal. B-Environ.* **2021**, *293*, 120216.

[18] T. Wu, X. Zhu, Z. Xing, S. Mou, C. Li, Y. Qiao, Q. Liu, Y. Luo, X. Shi, Y. Zhang, X. Sun, *Angew. Chem. Int. Ed.* **2019**, *58*, 18449.

[19] Y. Li, Q. Zhang, C. Li, H.-N. Fan, W.-B. Luo, H.-K. Liu, S.-X. Dou, *J. Mater. Chem. A* **2019**, *7*, 22242.
